# Antibiotic resistance and virulence genes profiling of *Vibrio cholerae* and *Vibrio mimicus* isolates from some seafood collected at the aquatic environment and wet markets in Eastern Cape Province, South Africa

**DOI:** 10.1371/journal.pone.0290356

**Published:** 2023-08-24

**Authors:** Oluwatayo E. Abioye, Nolonwabo Nontongana, Charles A. Osunla, Anthony I. Okoh

**Affiliations:** 1 Department of Microbiology, Obafemi Awolowo University, Ife, Nigeria; 2 SAMRC Microbial Water Quality Monitoring Centre, University of Fort Hare, Alice, South Africa; 3 Department of Microbiology, Adekunle Ajasin University, Akungba Akoko, Nigeria; University of Connecticut, UNITED STATES

## Abstract

The current study determines the density of *Vibrio* spp. and isolates *V*. *cholerae* and *Vibrio mimicus* from fish-anatomical-sites, prawn, crab and mussel samples recovered from fish markets, freshwater and brackish water. Virulence and antibiotic resistance profiling of isolates were carried out using standard molecular and microbiology techniques. *Vibrio* spp. was detected in more than 90% of samples [134/144] and its density was significantly more in fish than in other samples. *Vibrio*. *cholerae* and *V*. *mimicus* were isolated in at least one sample of each sample type with higher isolation frequency in fish samples. All the *V*. *cholerae* isolates belong to non-O1/non-O139 serogroup. One or more *V*. *cholerae* isolates exhibited intermediate or resistance against each of the eighteen panels of antibiotics used but 100% of the *V*. *mimicus* were susceptible to amikacin, gentamycin and chloramphenicol. *Vibrio cholerae* exhibited relatively high resistance against polymyxin, ampicillin and amoxicillin/clavulanate while *V*. *mimicus* isolates exhibited relatively high resistance against nitrofurantoin, ampicillin and polymixin. The multiple-antibiotic-resistance-index [MARI] for isolates ranges between 0 and 0.67 and 48% of the isolates have MARI that is >0.2 while 55% of the isolates exhibit MultiDrug Resistance Phenotypes. The percentage detection of *acc*, *ant*, *drf*18, *sul1*, *mcr-1*, *blasvh*, *blaoxa*, *blatem*, *blaoxa*48, *gyrA*, *gyrB* and *parC* resistance-associated genes were 2%, 9%, 14%, 7%, 2%, 25%, 7%, 2%, 2%, 32%, 25% and 27% respectively while that for virulence-associated genes in increasing other was *ace* [2%], *tcp* [11%], *vpi* [16%], *ompU* [34%], *toxR* [43%], *rtxC* [70%], *rtxA* [73%] and *hyla* [77%]. The study confirmed the potential of environmental non-O1/non-O139 *V*. *cholerae* and *V*. *mimicus* to cause cholera-like infection and other vibriosis which could be difficult to manage with commonly recommended antibiotics. Thus, regular monitoring of the environment to create necessary awareness for this kind of pathogens is important in the interest of public health.

## 1. Introduction

*Vibrio cholerae* strains that cause cholera epidemics and pandemics are found in marine and freshwater environments [1]. *Vibrio mimicus* is a close relative of *V*. *cholerae* and it causes gastroenteritis with symptoms similar to that of cholera. The vehicles of transmission of the two bacteria are water, fish and seafood contaminated with the bacteria [2–5]. The effective dose of *V*. *mimicus* for causing human infection is believed to be between 10^4^–10^6^ similar to that of *V*. *cholerae* which is between 10^3^–10^6^ [3]. *Vibrio mimicus* and *V*. *cholerae* have been isolated from the aquatic environment and seafood across the globe [3, 6–10]. They cause economic losses in aquaculture and mariculture globally [8, 10–12]. There have been seven cholera pandemics to date. The first six affected all the continents of the world while the ongoing seventh cholera pandemic is restricted to Africa, Asia and the Americas [13, 14]. Recent findings show that some geographical areas of Africa and Asia continents have become the homeland for cholera and the infection is believed to spread from there to other parts of the world as a result of migration to more urban areas and international travel [15].

The aquatic environment is the reservoir of *V*. *cholerae* [7, 16, 17]. Therefore, surveillance of the aquatic faunal and flora for *V*. *cholerae* and possibly other important human pathogenic *Vibrio* spp. such as *V*. *mimicus* in Asia and Africa countries is relevant for empirically based awareness creation and necessary restrictions which can help in preventing cholera and vibriosis outbreak. The work of [18] recommended the monitoring of the environment for *V*. *cholerae* when cholera outbreak is not ongoing and this could be extended to other human vibrio pathogens for cholera and vibriosis preventive purposes.

Seafood provides good protein for humans however, several kinds of seafood contaminated with pathogenic *Vibrio* spp. are responsible for major foodborne illnesses globally [9, 19, 20]. The practice of aquaculture and mariculture in the Eastern Cape Province contributes to South Africa’s GDP while commercial and recreational angling are peculiar to the rural and urban areas of the Province [21–23]. Pathogenic human *Vibrio* spp. have been reported from numerous surface water and wastewater treatment plants in Eastern Cape Province [24–29], however, there is a paucity of information about these pathogens in seafood from surface water resources and fish markets in the province. Of the only two studies on seafood from the province identified in the literature, the work of [30] did not detect *V*. *cholerae* and *V*. *mimicus* in their fish samples while [31] isolated the two pathogens but the report was only on mollusc.

Eastern Cape province is one of the provinces with high cases of reported cholera in South Africa between 2001 and 2003. The case fatality rate of 1.93 for the 2001–2002 outbreak and 1.18 for the year 2003 were higher than the average case fatality rate of the country [32]. Going by this peculiarity of the province, the aquatic animals in the province’s surface water resources and aquaculture markets could be harbouring pathogenic *Vibrio* spp. To investigate this hypothesis, the current study isolated *V*. *cholerae* and *V*. *mimicus* from fish, crab, mud brawn and mussel samples recovered from anglers and seafood markets in Eastern Cape Province when cholera outbreak is not ongoing. The antibiotic resistance profile and virulence capability of the isolates were also investigated.

## 2. Materials and methods

### 2.1 Samples and sampling sites

Sampling sites included two fish markets (one in East London and the other in Port Alfred), three freshwater and five brackish water resources. The water resources are used for both commercial and recreational angler activities. Samples were purchased from fish markets and anglers based on availability once a month for one year. Licensed anglers were identified at the water resources during a reconnaissance visit. The arrangement was made with them such that samples were recovered in our presence and immediately transferred into a sterile zip-lock bag. We also arranged with the fish market owners such that samples were purchased as soon as they got back from their fishing trip. Samples were purchased whole except in a few instances when fish heads and intestines have been removed by fishermen before returning from fishing trips. After purchase, samples were transported to the laboratory on ice packs in cooler boxes within six hours of collection.

### 2.2 Sample processing, and *Vibrio* density determination

Samples preparation was carried out using Bacteriological Analytical Manual as a guide [33]. The vibrio density determination was done using 3 tubes by 5 tenfold serial dilutions Most Probable Number-Polymerase Chain Reaction (MPN-PCR) method and the presumptive vibrio isolation was carried out as described in one of our previous studies [31]. Briefly, each sample comprised pulled 10 to 12 pieces of the sample. At the laboratory, following aseptic rules, fish samples were dissected to remove different anatomical sites (gill, flesh, intestine and fins which were designated as G, FL, IN and FN respectively) and the mussel sample was deshelled. Each of the pulled sample types [gill, flesh, intestine, fins, deshelled mussel, crab and mud prawn] was mashed in separate sterile mortar and pestle. Afterwards, ten grams of the mashed samples were gently pummeled in 90 ml of a sterile Phosphate Buffer Saline Solution [PBS] in the conical flask to have the first tenfold diluted sample homogenate (10^−1^). Ten millilitres of the first tenfold serially diluted sample homogenate was added to another 90 mL sterile PBS to have the second tenfold diluted sample homogenate (10^−2^). The process was repeated up to 10^−5^. One millilitre aliquot from the first tenfold diluted sample homogenate in the conical flask (10^−1^) was aseptically transferred into each of three separate test tubes containing sterile alkaline peptone water. The procedure was carried out for the reaming four tenfold serially diluted sample homogenate. The set-up (fifteen tubes per sample) was incubated at 37 °C for 24 hrs. and after the incubation period, total genomic DNA was extracted from turbid tubes using the boiling method as described elsewhere [34]. The total genomic DNA from each turbid tube was subjected to PCR (25 μl reaction) to detect the presence of *Vibrio* spp. in the turbid MPN tubes using a vibrio genus-specific primer used elsewhere [29]. The *Vibrio* spp. positive tubes were noted and the equivalent density of *Vibrio* spp. density was extrapolated from the MPN BAM-Excel sheet [35]. The Gene Technology G-Storm GS1 Thermal Cycler PCR Machine, USA was used for PCR and all media used in this study are Oxoid Cambridge, UK products. One Taq 2X Master Mix Standard Buffer (BioLabs, UK) and primers were supplied by Inqaba Biotec, South Africa. The details of the primer are given in S1 Table. Note that tissue e.g. gill from a fish sample [made up of 10–12 pieces of the same fish] purchased at a sampling site formed one gill sample and this applies to other anatomical sites as well.

### 2.3 Isolation of presumptive *Vibrio* spp. their PCR confirmation and delineation

Ten grams of the sample prepared in section 2.2 above were enriched with 90 ml of APW and incubated at 37 °C for 24 hrs. After the incubation period, a loop full of the APW directly underneath the surface pellicle formed was streaked on freshly prepared Thiosulfate–citrate–bile salts–sucrose agar (TCBS, Oxoid Cambridge, UK) and incubated for 24 hrs. After the incubation period, two to ten green and yellow distinct colonies that represent the different colony morphology observed on the TCBS plate were selected as presumptive *V*. *cholerae* and *V*. *mimcius* isolates respectively. The presumptive isolates were purified by streaking them on sterile 1% NaCl nutrient agar plates, checking the morphological uniformity of colonies along the line of streak coupled with Gram-staining. The presumptive isolates were confirmed as *Vibrio* spp. using the vibrio genus-specific primers and confirmed isolates were afterwards delineated into *V*. *cholerae* and *V*. *mimicus* using species-specific primers in a 25 μl PCR reaction. Furthermore, *V*. *choleae* isolates were screened for the presence of O1 and O139 strains using specific primers that targeted *rfb* regions responsible for “O” antigen biosynthesis [36, 37]. The details of the primers and amplification conditions are given in S1 Table.

### 2.4 Antibiotics susceptibility testing and detection of virulence and antibiotics resistance genes

The antibiotic susceptibility testing was performed by Kirby-Bauer Disk Diffusion Susceptibility Test Protocol [38] and interpreted according to the recommendation of the Clinical Standard Institute (CLSI) [39]. The panel of antibiotics used for the study is as recommended by CLSI and those that have been used against *Vibrio* isolates in previous studies [24, 25, 29, 40–43]. The antibiotics are: kanamycin (K) 30 μg, amikacin (AK) 30 μg, gentamycin (GM) 4 μg, meropenem (MEM) 10 μg, imipenem (IMI) 10 μg, cefotaxime (CTX) 30 μg, cefuroxime (CXM) 30 μg, ofloxacin (OFX) 5 μg, ciprofloxacin (CIP) 5 μg, Nalidixic acid (NA) 30 μg, Azithromycin (ATH) 15 μg, Nitrofurantoin (NI) 300 μg, Amoxicillin/clavulanate (AUG) 30 μg, Ampicillin (AP) 10 μg, chloramphenicol (C) 30 μg, Polymyxin B (PB) 300 units, cotrimoxazole (TS) 25 μg and trimethoprim (TM) 5 μg. Multiple antibiotics resistance index (MARI) was calculated and interpreted for each isolate, sample type and sampling area as recommended. Isolates, sample type and sampling area with ≥ 0.2 MARI were identified as agents/sources of high risk of antibiotic-resistant vibrio infection [44]. The MARI was calculated using Eq 1 below. *E*. *coli* ATCC 25922 was used as the quality control organism.

MARIforanisolate=a/b (1)

where a = number of antibiotics an isolate exhibited resistant against and b = the total number of antibiotics tested against the isolate.

Multiple antibiotics resistance phenotypes (MARP) i.e. isolates that exhibited resistance against more than one antibiotic and multidrug antibiotics resistance phenotypes (MDRP) i.e. isolates that exhibited resistance against at least one antibiotic in three or more classes of antibiotics that were used for this study [45]. Isolates based on their phenotypic resistance profile were tested for the presence of some antibiotic resistance genes [*cmlA*, *cat*, *flor*, *aac*, *ant*, *aphA*, *drf18*, *sul1*, *sul 2*, *mcr-1*, *blas*_*shv*_, *bla*_*oxa*_, *bla*_*tem*_, *bla*_*vim*_, *bla*_*kpc*_, *bla*_*ndm*_, *bla*_*imp*_, *bla*_*oxa48*_, *gyrA*, *gyrB*, *parc*, *sxt*] in a 25 μl PCR reaction.

Also, to understand the pathogenicity of the confirmed isolates, they were tested for the presence of eleven virulence determinants (details in S1 Table) in a 25 μl PCR reaction. The virulence genes selected are those that have been implicated in cholera and vibriosis caused by *V*. *cholerae* and *V*. *mimicus*. The multi-virulence gene indexes (MVGI) and the multi-resistance gene indexes of the isolates were also determined using a method reported elsewhere [46]. The antibiotics-resistant genes targeted, primers details and thermal conditions used for all PCR reactions are given in S1 Table.

### 2.5 Statistics

The *Vibrio* spp. mean density, MARI, MRGI, MVGI and MDRP was subjected to a normality test using Kolmogorov-Smirnov and Shapiro-Wilk test to determine whether to use a parametric or non-parametric test. Afterwards, an appropriate statistical test was employed to compare the *Vibrio* spp. density across sample types while the relationship between MARI, MVGI, MRGI and MDRP was determined using an appropriate correlation test. Correlation analysis between MARI, MVGI, MRGI and MDRP was carried out to observe the type of relationship that exists between phenotypic resistance, prevalence of virulence determinants, prevalence of resistance genes and prevalence of multidrug-resistant phenotypes. The differences between the magnitude of antibiotics resistance exhibited [using MARI as the corresponding variable], prevalence of virulence determinants (using MVGI as the corresponding variable) and prevalence of antibiotics resistance genes (using MRGI as the corresponding variable) across the *Vibrio* spp. (*V*. *cholera* and *V*. *mimcicus*) isolates, sampling sites, sample types, sample sources and sample class were also statistically determined. The significant level was set at <0.05. SPSS version 25 was used for the statistical analysis.

## 3. Result

### 3.1 *Vibrio spp*. density and distribution of *V*. *cholerae* and *V*. *mimicus* in samples

**Fig 1:**
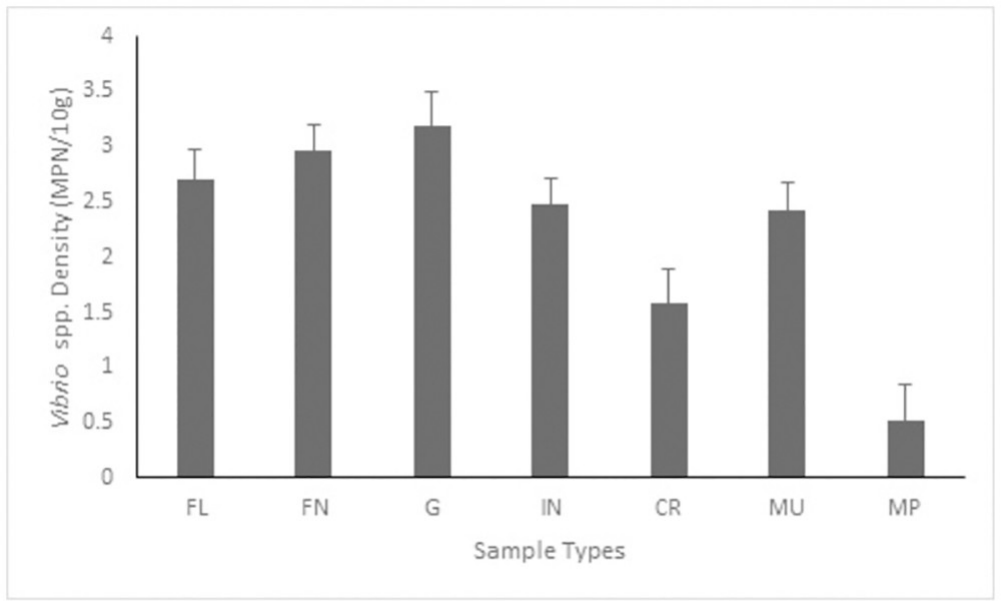
Average *Vibrio* spp. density per sample type. Key: FL = flesh, FN = fin, G = gill, IN = intestine.

The mean *Vibrio* spp. density across sample types is given in Fig 1 with gill having the highest mean density while mud prawn has the lowest. The density of *Vibrio* spp. for each sample type and site is given in Fig 1 and Table 1. A total of 29 fish samples which resulted in 29 flesh, 24 fins, 26 gills and 18 intestine samples were recovered from the fish markets and surface water. The crab, mud prawn and mussel samples collected from surface water were 24, 9 and 14 respectively. Of the 17 samples recovered from the fish markets, 41% were positive for one or both *V*. *cholerae* and *V*. *mimicus*. On the other hand, 25% (3/12) of the fish samples recovered from the surface water were positive for the two *Vibrio* species. At least one of the anatomical sites of all the fish samples was positive for *Vibrio* spp. however, *Vibrio* spp. was not isolated in 3 fins, 4 flesh, 2 intestine and one gill samples. Only 27.59% [8/29] of the fish samples (2 flesh, 3 fin, 2 gill and 2 intestine samples) were positive for *V*. *cholera*e while 10.34% (3/29) of the fish samples (2 gill and 2 intestine samples) were positive for *V*. *mimicus*. None of the mud prawn samples was positive for *V*. *mimicus* but 30% (3/10) of the samples were positive for *V*. *cholera*. On the other hand, 7.14% (1/14) and 14.29% (2/14) of the mussel samples were positive for *V*. *mimicus* and *V*. *cholera* respectively. Furthermore, 12.50% (3/24) and 4.17% (1/24) of the crab samples were positive for *V*. *mimicus* and *V*. *cholera* respectively. Of the 535 presumptive isolates (FL = 145, FN = 114, G = 165 and IN = 111) recovered from the fish samples, 81% (434/535 [FL = 119, FN = 93, G = 124 and IN = 98]) were confirmed as *Vibrio* spp. and 3% (21/434 [FL = 7, FN = 8, G = 3 and IN = 3) and 1% (4/434 [FL = 0, FN = 0, G = 2 and IN = 2]) were confirmed as *V*. *cholerae* and *V*. *mimicus* respectively. Given the above, the prevalence of *V*. *cholera* among the population of confirmed *Vibrio* spp. from the flesh, fin, gill and intestine samples were 5.88%, 8.60%, 2.42% and 3.06% respectively while the prevalence of *V*. *mimicus* was 0%, 0%, 1.61% and 2.04% respectively. Seventy-four presumptive isolates were recovered from crab samples out of which 68% (51/75) were confirmed as *Vibrio* spp. while approximately 11% (8/75) and 3% (2/75) isolates of the confirmed *Vibrio* spp. were *V*. *cholerae* and *V*. *mimicus* respectively. The presumptive isolates from mud prawn samples were fifty out of which 76% (38/50) were confirmed as *Vibrio* spp. An 8% (3/38) and 0% (0/38) of the population of the confirmed *Vibrio* spp. were *V*. *cholerae* and *V*. *mimicus* respectively. The presumptive isolates recovered from mussel samples were 211 of which 92% (195/211) were confirmed as members of the vibrio genus. Of those confirmed, 1% (2/195) and 2% (4/195) were confirmed as *V*. *cholerae* and *V*. *mimicus* respectively. A total of 33 and 9 *V*. *cholerae* and *V*. *mimicus* respectively were recovered from samples and most of the isolates 59% (26/44) were recovered from fish anatomical sites. All the *V*. *cholerae* were confirmed to belong to non-O1/non-O139 serogroup.

**Table 1:** Percentage prevalence of *Vibrio* spp., *V*. *cholerae* and *V*. *mimicus* in sample types and at sampling sites.

**Var**	**FISH TYPES [n]**															
	**Ba (3)**	**Br (6)**	**CAP (1)**	**CAT (2)**	**Eel (1)**	**KOB (1)**	**MA (2)**	**MAC (2)**	**ML (1)**	**PI (2)**	**RER (2)**	**SN((1)**	**TF (3)**	**WH (1)**	**YT (1)**	**% Total**
**%16S**	95	82	22	92	100	81	61	60	96	75	50	80	88	100	70	81
**%Vc**	0	12	0	0	17	10	11	0	0	0	0	0	9	0	0	5
**%Vm**	0	1	0	0	0	0	0	0	0	0	0	7	0	8	0	1
**Var**	**Other sample types (n)**			**Var**				**Sampling sites (n)**								
	**CR (24)**	**MU (14)**	**MP (10)**					**Fish Market (16)**			**Brackish Water (49)**			**Fresh Water (12)**		
**%16S**	69	92	76	**%16S**				68			86			94		
**%Vc**	16	1	8	**%Vc**				8			4			6		
**%Vm**	4	2	0	**%Vm**				2			1			1		

Key: Var = Variable, %16S = percentage of recovered isolates that were confirmed as *Vibrio* spp., %Vc = % of the confirmed *Vibrio* spp. that were *V*. *cholera*, %Vm = percentage of the confirmed *Vibrio* spp. that are *V*. *mimicus*, Ba = Bass Fish, Br = Bream Fish, Cap = Carpenter Fish, El = Eel Fish, KOB = Duskykob, MA = Maasbanker Fish, MAC = Mackerel Fish, ML = Mullet Fish, PI = Pilchards Fish, RER = Red Roman Fish, SN = Snok Fish, TF = Tiger Fish, WH = White Fish, YT = Yellow tail Fish, CR = Crab, MU = Mussel, MP = Mud prawn.

### 3.2 Phenotypic and genotypic antibiotics resistance profile of *V*. *cholerae* and *V*. *mimicus* isolates and detection of virulence genes

**Fig 2:**
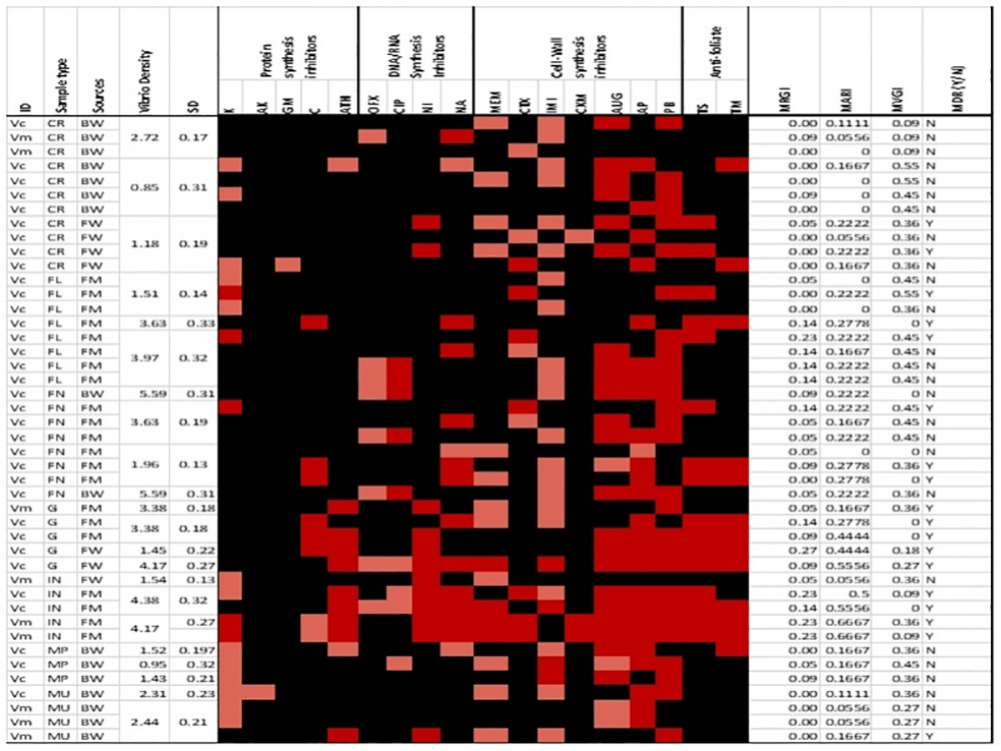
Phenotypic antibiotics susceptible, intermediate and resistance patterns of *V*. *cholerae* and *V*. *mimicus* isolates. Key: ID = identity, (FL = flesh, FN = fin, G = gill, IN = intestine), MU = mussel, MP = mud prawn samples, ARP = antibiotics resistance profile, MARI = multiple antibiotic resistance index, MDR = multi-drug resistance status, Y = yes, N = No, black = susceptible, pink = intermediate and red = resistance, MVGI = Multi-virulence gene Index, MRGI = Multi-resistance gene index.

The percentage prevalence of isolates that exhibit susceptible, intermediate and resistance phenotypic characteristics is given in Table 2 while the prevalence of virulence determinants and antibiotics resistance determinates are given in Table 3. The phenotypic antibiotics susceptible, intermediate and resistance patterns, MARI, MVGI, MRGI and MDR status of the isolates are given in Fig 2.

**Table 2:** Percentage prevalence of *V*. *cholerae* and *V*. *mimicus* that exhibited susceptible, intermediate and resistance phenotypic characteristics against panels of antibiotics.

Classes	Antibiotics	*V*. *cholera*			*V*. *mimicus*		
		S (%)	I (%)	R (%)	S (%)	I (%)	R (%)
2nd generation Cephaloporin	Cefuroxime (CXM) 30 μg	97.1	2.9	0.0	77.8	0.0	22.2
3rd generation Cephaloporin	Cefotaxime (CTX) 30 μg	77.1	8.6	14.3	66.7	11.1	22.2
Aminoglycoside	Kanamycin (K) 30 μg	62.9	28.6	8.6	44.4	33.3	22.2
	Amikacin (AK) 30 μg	97.1	2.9	0.0	100.0	0.0	0.0
	Gentamycin (GM) 4 μg	97.1	2.9	0.0	100.0	0.0	0.0
Antifoliate	Trimethoprim (TM) 5 μg	68.6	0.0	31.4	77.8	0.0	22.2
Antifoliate/sulfonamide combination	Cotrimoxazole (TS) 25 μg	60.0	0.0	40.0	77.8	0.0	22.2
Carbapenems	Meropenem (MEM) 10 μg	68.6	25.7	5.7	44.4	33.3	22.2
	Imipenem (IMI) 10 μg	37.1	51.4	11.4	66.7	33.3	0.0
Fluroquinolones	Ofloxacin (OFX) 5 μg	80.0	20.0	0.0	88.9	11.1	0.0
	Ciprofloxacin (CIP) 5 μg	74.3	11.4	14.3	100.0	0.0	0.0
Quinolone	Nalidixic acid (NA) 30 μg	65.7	8.6	25.7	66.7	0.0	33.3
Macrolide	Azithromycin (ATH) 15 μg	80.0	5.7	14.3	55.6	0.0	44.4
Nitrofuran [Microbid]	Nitrofurantoin (NI) 300 μg	80.0	0.0	20.0	44.4	0.0	55.6
Phenicol	Chloramphenicol (C) 30 μg	82.9	0.0	17.1	77.8	22.2	0.0
Polypeptide	Polymyxin B (PB) 300 units	31.4	0.0	68.6	55.6	0.0	44.4
β-lactam	Ampicillin (AP) 10 μg	37.1	2.9	60.0	55.6	0.0	44.4
β-lactam/β-lactamase inhibitor combination	Amoxicillin/clavulanate (AUG) 30 μg	37.1	5.7	57.1	55.6	22.2	22.2

**Table 3:** Percentage prevalence of detected antibiotics resistance genes and virulence determinants among *V*. *cholerae* and *V*. *mimicus* population.

Antibiotics resistance genes (%)											
Organisms	*ant*	*Drf 18*	*blasSHV*	*sul 1*	*gyrA*	*gyrB*	*ParC*	*mrc-1*	*blaoxa*	*blaTem*	*blaoxa48*
*V*. *cholerae*	11.4	11.4	2.9	2.9	22.9	14.3	20.0	2.9	2.8	8.6	2.9
*V*. *mimicus*	ND	ND	ND	ND	22.2	11.1	ND	ND	22.22	ND	ND
Virulence genes (%)											
Organisms	*vpi*	*toxR*	*ompU*	*Tcp*	*Ace*		*hyla*		*rtxA*		*rtxC*
*V*. *cholerae*	17.1	54.3	37.1	11.4	2.9		74.3		74.3		71.4
*V*. *mimicus*	11.1	0.0	22.2	11.1	0.0		88.9		66.7		66.7

Above 50% of all the isolates were susceptible to kanamycin, amikacin, gentamycin, meropenem, imipenem, cefotaxime, cefuroxime, ofloxacin, ciprofloxacin, nalidixic acid, azithromycin, nitrofurantoin and chloramphenicol. Over fifty per cent of isolates exhibited resistance against polymyxin B, amoxicillin/clavulanate and ampicillin while 20% to 36% of the isolates exhibited resistance against nalidixic acid, azithromycin, nitrofurantoin, cotrimoxazole and trimethoprim. Eighteen per cent, 30% and 48% of the isolates exhibited intermediate characteristics against ofloxacin, kanamycin and imipenem respectively. The MARI for the isolates ranges between 0 and 0.67 and 48% of the isolates have MARI that is above 0.2 while 55% of the isolates exhibit MDR phenotypes.

Of the 22 resistant determinants targeted in this study, 12 (55%) were detected in at least one of the isolates. The percentage detection of *acc*, *ant*, *drf*18, *sul1*, *mcr-1*, *blasvh*, *blaoxa*, *blatem*, *blaoxa*48, *gyrA*, *gyrB* and *parC* were 2%, 9%, 14%, 7%, 2%, 25%, 7%, 2%, 2%, 32%, 25% and 27% respectively. Furthermore, 8 [73%] of the eleven virulence determinants targeted in this study were detected in at least one of the isolates. The percentage detection of the virulence-associated genes in increasing other was *ace* (2%), *tcp* (11%), *vpi* (16%), *ompU* (34%), *toxR* (43%), *rtxC* (70%), *rtxA* (73%) and *hyla* (77%). Of the eight virulence-associated genes detected, *toxR* and *ace* genes were not detected in *V*. *mimicus* but *vpi*, *OmpU tcp*, *hyla*, *rtxA* and *rtxc* were detected in 11%, 22% 11%, 89%, 67% and 67% of the *V*. *mimicus* isolates. The percentages of *V*. *cholreae* isolates that carry the eight genes (*vpi*, *toxR*, *OmpU tcp*, *ace*, *hyla*, *rtxA* and *rtxc*) were 17.14, 54.29, 37.14, 11.43, 2.86, 74.29, 74.29 and 71.43 respectively. Representative gel pictures showing products of the amplification of the region targeted for each virulence determinant are given in S1–S5 Figs while that for the resistance determinants are given in S6–S8 Figs.

### 3.3 Resistance-associated genes, virulence-associated genes and phenotypic resistance combinations detected in isolates

The highest number of virulence genes detected concurrently in a *V*. *cholerae* isolate was 6 and this is found in just one isolate (Table 4). The highest number of the virulence determinants detected simultaneously in a singular *V*. *mimicus* isolate per time was four and this was observed in three *V*. *mimicus* isolates (Table 4). The combinations of virulence genes, antibiotic resistance genes and phenotypic resistance observed are given in Table 4. A total of fourteen different combinations of the virulence genes targeted were detected. Only one virulence combination type was common to both *V*. *cholerae* and *V*. *mimicus* isolates while nine combinations were only found among the *V*. *cholera* population, four were found only among *V*. *mimicus* population.

**Table 4:** Resistance-associated genes, virulence-associated genes and phenotypic resistance profiles of isolates.

*Vibrio cholera* [non-O1/non-O139]					
Isolates	STs	Sources	Resistance Genes	Phenotypic Resistance	Virulence genes
816	MP	Brackishwater	ant	IMI,PB,AP	_
564	FL	Market	Drf18,blasSHV,gyrA,gyrB,ParC	K,CTX,PB,TS	_
778	FL	Market	ParC	_	_
783	FL	Market	_	_	_
561	FN	Market	Drf18,blasSHV,gyrA	K,CTX,PB,TS	_
562	FN	Market	blasSHV	NA,PB,AUG	_
798	FN	Market	ParC	_	_
279	CR	Freshwater	_	PB,AUG	hyla
563	FN	Market	blasSHV	PB,CIP,AUG,AP	hyla
614	CR	Brackishwater	_	AUG,AP,TM	OmpU, hyla,rtxA, rtxC
615	CR	Brackishwater	_	PB,AUG	OmpU, hyla,rtxA, rtxC
617	CR	Brackishwater	gyrA, ParC	PB,AUG	OmpU, hyla,rtxA, rtxC
619	CR	Brackishwater	_	PB,AP	OmpU, hyla,rtxA, rtxC
233C	IN	Market	_	NA,MEM,ATH,IMI,PB,NI,TS,AUG,AP,TM	OmpU, hyla,rtxA, rtxC
982	FN	Market	gyrA, ParC	NA,TS,C,AP,TM	tcp, rtxA
983	FN	Market	_	AK,TS,C,AP,TM	tcp, rtxA, rtxC
938	FL	Market	sul1, gyrA, ParC	NA,TS,C,AP,TM	toxR, hyla, rtxA, rtxC
779	FL	Market	_	K,CTX,PB,TS	toxR, hyla, rtxA, rtxC
984	G	Market	sul1,gyrA,ParC	AK,A,C,AP,TM	toxR, hyla, rtxA, rtxC
252	MU	Brackishwater	_	PB,AP	toxR, OmpU, hyla,rtxA, rtxC
905	CR	Freshwater	Gent ant	PB,NI,TS,AUG	toxR, OmpU, hyla,rtxA, rtxC
906	CR	Freshwater	_	AP	toxR, OmpU, hyla,rtxA, rtxC
907	CR	Freshwater	_	PB,NI,TS,AUG	toxR, OmpU, hyla,rtxA, rtxC
565	FL	Market	blasSHV,gyrA,gyrB	NA,PB,AUG	toxR, OmpU, hyla,rtxA, rtxC
566	FL	Market	blasSHV,gyrA,gyrB	PB,CIP,AUG,AP	toxR, OmpU, hyla,rtxA, rtxC
567	FL	Market	blasSHV,gyrA,gyrB	PB,CIP,AUG,AP	toxR, OmpU, hyla,rtxA, rtxC
430	FN	Brackishwater	blaTem	PB,CIP,AUG,AP	toxR, tcp,ace,hyla,rtxA, rtxC
79C	G	Freshwater	mcr-1,gyrB	NA-MEM-ATH-IMI-PB-NI-TS-AUG-AP-TM	toxR, tcp,ace,hyla,rtxA, rtxC
184C	IN	Market	ant,Drf 18,blaSHV,gyrA,gyrB	NA-ATH-CTX-PB-NI-TS-AUG-AP-TM	vpi, toxR, hyla, rtxA, rtxC
985	G	Market	sul1,ParC	ATH-PB-NI-TS-C-AUG-AP-TM	vpi, toxR, hyla, rtxA, rtxC
60	MP	Brackishwater	_	AUG-AP-TM	vpi, toxR, hyla,rtxA, rtxC
817	MP	Brackishwater	ant,blaTem	IMI-PB-AUG	vpi, toxR, hyla,rtxA, rtxC
908	CR	Freshwater	_	CTX-AP-TM	vpi, toxR, hyla,rtxA, rtxC
12C	G	Freshwater	acc,Drf18, blaoxa, blaoxa48,gyrA,gyrB	ATH-PB-NI-TS-C-AUG-AP-TM	vpi, toxR, OmpU, hyla,rtxA, rtxC
433	FN	Brackishwater	blaTem, gyrB	PB-CIP-AUG-AP	vpi, toxR, tcp, hyla,rtxA, rtxC
*Vibrio mimicus*					
Isolates	STs	Sources	Resistance Genes	Phenotypic Resistance	Virulence genes
281	CR	Freshwater	_	ATH-PB-NI	hyla
920	CR	Freshwater	ParC	AP	hyla
147cC	G	Market	_	AP	OmpU, hyla, rtxA, rtxB
280	IN	Freshwater	gyrA, gyrB	NA	vpi, OmpU, hyla, rtxA
147aC	IN	Market	_	_	tcp, hyla, rtxA, rtxB
147bC	IN	Market	_	NI	hyla
502	MU	Brackishwater	gyrB	ATH-PB-NI	hyla, rtxA, rtxB
368aC	MU	Brackishwater	Drf18,blasSHV,blasoxa,gyrA,ParC	K-N-,MEM-ATH-CTX-PB-NI-TS-CXM-AUG-AP-TM	hyla, rtxA, rtxB
380C	MU	Brackishwater	Drf18,blasSHV,blasoxa,gyrA,ParC	K-NA-MEM-ATH-CTX-PB-NI-TS-CXM-AUG-AP-TM	hyla, rtxA, rtxB

Key: STs = Sample types; [FL = flesh, FN = fin, G = gill, IN = intestine], MU = mussel, MP = mud prawn sample,— = non detected, The antibiotics are:K = kanamycin, AK = amikacin GM = gentamycin, MEM = meropenem, IMI = imipenem, CTX = cefotaxime, CXM = cefuroxime, OFX = ofloxacin, CIP = ciprofloxacin, NA = Nalidixic acid, ATH = Azithromycin, NI = Nitrofurantoin [NI], AUG = Amoxicillin/clavulanate, AP = Ampicillin, C = chloramphenicol, PB = Polymyxin B, TS = cotrimoxazole and TM = trimethoprim, Ant = aminoglycoside nucleotidyltransferase gene, Drf 18 = trimethoprim-resistance genes, blaSHV = beta-lactamase [sulf-hydryl variable active site] gene, gyrA = gyrase A gene, gyrB = gyrase B gene, sul1 = sulfonamide resistance gene, ParC = gene encoding topoisomerase IV, blaTem = beta-lactamase, temoniera gene, acc = aminoglycoside 6′-N-Acyltransferase gene, blaoxa = oxacillinases gene, blaoxa48 = oxacillinases gene variant 48, OmpU = pore-forming proteins gene of the outer membrane, hyla = El Tor-specific haemolysin toxin gene, rtxA = acyltransferase gene, rtxC = presumptive cytotoxin gene, tcp = toxin co-regulated pilus gene, toxR = essential transcription factor gene, ace = accessory cholera enterotoxin gene, vpi = vibrio pathogenic island

Eleven resistance gene profiles were observed, one profile was common to both *V*. *cholerae* and *V*. *mimicus*, one was only found among *V*. *mimicus* population and nine among only the *V*. *cholera* isolates. The highest resistance genes detected concurrently among *V*. *cholerae* isolate were five and two for *V*. *mimicus*. Twenty-one resistance phenotypes were detected of which one was common to the two *Vibrio* spp. while sixteen were found among *V*. *cholerae* isolates only and four among the *V*. mimicus population only. The highest concurrent phenotypic resistance detected in a *V*. *cholerae* isolate was against ten panels of antibiotics while that for *V*. *mimicus* was against thirteen. The distribution of resistance genes, phenotypic resistance and virulence genes combinations (profiles) among *V*. *cholerae* and *V*. *mimicus* populations is given in S3 Table while the dynamics of resistance for sampling sites, sample types, sources of samples and classes of samples using MARI, MARP AND MDRP as indices is given in S4 Table.

### 3.4 Statistical analysis

The variables (*Vibrio* spp. mean density, MVGI, MRGI and MARI) failed Kolmogorov-Smirnov and Shapiro-Wilk tests and thus tools for non-parametric data were used for statistical analysis. Welch ANOVA test result revealed a significant difference in *Vibrio* spp. density across sample types F_[6, 46.37]_ = 7.62 P < 0.001. Game-Howell post Hoc analysis result given in Table 5 indicated that *Vibrio* species. density in gill, fin and flesh samples were significantly more than that in mud prawn and crab samples. Other comparisons of density across sample types were not significantly different. Spearman rho correlation analysis carried out determined the relationship between MARI, MVGI, MDRP and MRGI because the four variables failed Kolmogorou-Smirnov and Shapiro-Wilk tests and for the same reason, the Wilcoxon test was used to compare the differences between the three variables (MARI, MVGI and MRGI) across *Vibrio* spp. while Welch ANOVA and Game-Howell post hoc analysis was used to compare the three variables across sampling sites, sample types, sample sources and sample classes. ANOVA was adopted for the interpretation where Welch tests cannot be performed due to a group having a variance of zero. The result of the correlation test (Table 6) revealed significant positive or negative relationships between variables except for MRGI vs MVGI which is negative but not significant. The inverse relationship between levels of antibiotics resistance (determined by MARI, MRGP and MRGI) among isolates and the prevalence of virulence determinants (determined by MVGI) observed in this study have been reported in earlier studies [47, 48] and it has been shown that the inverse relationship is as a result of fitness trade-off [49]. However, a study [50] has reported no correlation between the variables while another one [51] observed a direct relationship. These observations confirmed a complex relationship between the detection of virulence and antibiotic resistance. To unravel this complexity, [52] carried out a review study and found out that the interplay between virulence, antibiotics resistance and the associated biological costs depends on four main factors which are the bacterial species involved, virulence and resistance mechanism at play, the ecological niche involved, and the host in question. Wilcoxon test showed that the MARI and MRGI of *V*. *mimicus* compare to that of *V*. *cholerae* were not significantly different but their MVGI were significantly different. The statistical comparison [Table 7] showed that MARI and MRGI of *V*. *cholerae* and *V*. *mimicus* showed varying levels of significant differences across sampling sites, sample types, sample sources and sample class while MVGI is not significantly different across the aforementioned variables.

**Table 5:** Excerpt from Game-Howell Pot Hoc analysis showing sample types with significantly different *Vibrio* spp. density.

[I] sample types	[J] sample types	Mean Difference [I-J]	Std. Error	P values	95% Confidence Interval	
					Lower Bound	Upper Bound
G	Cr	1.60858*	0.35	0.00	0.53	2.68
	MP	2.01152*	0.40	0.00	0.75	3.27
FN	Cr	1.38021*	0.36	0.01	0.27	2.49
	MP	1.78316*	0.40	0.00	0.49	3.07
FL	Cr	1.11111*	0.32	0.02	0.11	2.11
	MP	1.51405*	0.38	0.01	0.31	2.72

Key G = gill, FN = Fin, FL = Flesh, Cr = Crab,

**Table 6:** Correlation analysis to determine the relationship between MARI, MVGI, MRGI and MDRP [n = 44].

Variables	MARI	MVGI	MRGI
MARI	1		
MVGI	-.299*	1	
MRGI	.626**	-0.151	1
MDRP	.754**	-.321*	.422**

Key: MARI = multiple antibiotics resistance index, MVGI = multiple virulence gene index, MRGI = multiple resistance gene index, MDRP = multiple drug resistance phenotypes, n = number of isolates

**Table 7:** The statistically significant differences of MARI, MRGI and MVGI across sampling sites, sample types, source of sample and sample class.

Sampling site (n = 44)	Sample types (n = 44)	Sample sources (n = 44)	Sample class (n = 44)
MARI*^a^	MARI*^b^	MARI*^a^	MARI*^a^
MVGIa	MVGI a	MVGIa	MVGIa
MRGI*^a^	MRGI*^b^	MRGI*^a^	MRGI*^b^
MARI*b	MARIb	MARI*^b^	MARIa
MRGI*b	MRGIb	MRGIb	MRGIa
MRGIb	MRGIb	MRGIb	MRGIa

Key:

* = significantly different,

^a^ = interpretation by welch and

^b^ = interpretation by ANOVA.

## 4. Discussion

### 4.1 *Vibrio* spp. density and the prevalence of *V*. *cholerae* and *V*. *mimicus* in samples

The density of *Vibrio* spp. differs numerically across anatomical sites of fish and other aquatic animals studied. The mean density of the *Vibrio* spp. in the fish anatomical sites except intestinal samples was significantly more than that of the crab and mussel samples. The gill sample has the highest average *Vibrio* spp. density and this is in concordance with an earlier study [53, 54]. The relatively high occurrence of *V*. *cholera* and *V*. *mimicus* in fish samples when compared to other samples supports earlier studies that reported that fish is one of the main reservoirs of *V*. *cholera* and *V*. *mimicus* in the aquatic environment. [1, 20, 55–58]. The low prevalence of *V*. *cholerae* and *V*. *mimicus* amidst the total number of confirmed *Vibrio* spp. recovered in this study is similar to earlier studies [55, 56]. This could be due to the largeness of the *Vibrio* genus which is over 100 species [59].

Cholera-causing *V*. *cholerae* serotypes (O1 and O139) are not commonly isolated from seafood as observed in this study nevertheless, the non-O1/non-O139 serogroups isolated have been implicated in human and aquatic animal infections outbreaks [60–64]. The relatively high prevalence of *V*. *cholerae* and *V*. *mimicus* in market samples than in surface water samples suggests a higher risk of contracting non-O1/non-O139 *V*. *cholera* and *V*. *mimicus* infections at the fish market in Eastern Cape than at the surface water. As relayed by the owners, the fish sold at the fish markets used for this study are from outside Eastern Cape Province. This calls for a more stringent microbial examination of fish products imported into the province.

### 4.2 The complexity and diversity of virulence determinants mix in non-O1 and non-O139 *V*. *cholerae* and *V*. *mimicus* isolates and implication

The current study shows the potential complexity and variation that could be observed in the virulence determinant profile of non-O1/non-O139 strains isolated from a similar geographical location. Horizontal gene transfers and serotype conversion has been linked to this observation. All four *tcp*^+^ non-O1/non-O139 isolates were *rtx*^*+*^ while two were *toxR*^+^. One of the *toxR*^+^ isolates was *hylA*^*El Tor*^ positive while the other was positive for *vpi*.

The *ace* gene, a core gene of the CTX elements which encodes enterotoxin detected in one of the isolates, is rarely found in non-O1/non-O139 strains but when detected, it is as a result of serogroup conversion of non-pathogenic environmental strains through horizontal gene transfer mechanisms to pathogenic ones [65]. The *tcpA* gene which is another core CTX element that encode the major structural unit of toxin-coregulated pilus detected in this study is believed to be a specific O1 and O139 serogroup virulence gene. However, the possibility of the acquisition of the *tcp A* gene by non-O1/non-O139 via horizontal gene transfer has been posited [66] and this explains its detection in the current study and an earlier study.

The VPI gene detected in 17% of the isolates in this study is an essential virulence gene cluster that enhances the colonization of the human intestine as well as the activity of the infection receptor site for most toxigenic Vibrio strains [67]. The toxin coregulated pilus [TCP], an essential colonization factor, is encoded within the VPI and the highland also encodes several virulence regulators and sialic acid utilization genes [68, 69]. All the *vpi-*positive isolates in our study harboured the two *rtx*, *toxR*, *hylA*^*El Tor*^ and one of them tested positive for *tcpA* gene as earlier reported [70]. On the other hand, *tcpA*^+^, *toxR*^+^ *vpi*^-^ strains were detected and this is in line with studies that reported *Vibrio cholerae* strain that is positive for cholera toxin but negative for vibrio pathogenicity highland [71, 72] and *Vibrio alginolyticus*, *V*. *cholerae* O1, O139 and non-O1/non-O139 strains that are *vpi* negative but *tcpA* positive [70, 73]. Genomic sequence and comparative studies have confirmed the inter-specific and intra-specific differences in the content of the pathogenicity highlands. Also, it has been reported that the three *Vibrio cholera* highlands can excise from the chromosome and circular intermediates and VPI can be transferred from *vpi+* strain to *vpi-* strain [74–77].

As usually reported, *OmpU*^*+*^ *V*. *cholerae* isolates are also *ToxR*^*+*^ since *ToxR* is needed for the regulation of *OmPU* gene [78–82]. However, only 61.5% of the *OmpU*^*+*^of non-O1/non-O139 isolates were *ToxR*^*+*^ and the two *OmpU*^*+*^ *V*. *mimicus* also lack the *ToxR* gene in this study. *OmpU*^*-*^*ToxR*^*-*^ and *OmpU*^*-*^*ToxR*^*+*^ *V*. *cholerae* isolates have been reported [78–83] but *OmpU*^*+*^ *ToxR*^*-*^ strains are here reported for the first time. We suspected deletion or outright absence of the *ToxR* gene in the *OmpU*^*+*^ *ToxR*^*-*^ strains since *ToxR* gene could be chromosomal or plasmid-borne and deletion/acquisition of the gene has been reported [84–90]. The *OmpU* gene can still be expressed in the presence of the *ompT* promoter and the absence *ToxR* gene [88]. Although we did not target *ToxT* gene in this study the aforementioned explained the possibility of *OmpU*^*+*^ and *ToxR*^*-*^ genotypes in *Vibrio* species that will express *OmpU* in the absence of *ToxR* gene. None of the eleven virulence-associated genes was detected in 20% of the *V*. *cholerae* isolates in this study. The non-O1/non-O139 strain that lacks key structural and regulatory pathogenicity genes have been reported [65].

*Vibrio cholerae* virulence genes have been reported in other human vibrio pathogens such as *V*. *alginolyticus* and *V*. *mimicus* [65, 70]. All the virulence-associated genes detected in the *V*. *cholerae* isolates in this study were also detected in *V*. *mimicus* isolates except for *ToxR* and *ace* genes. The study carried out by [8] on the genomic comparison between environmental *V*. *mimicus* and clinical isolates concluded that the genes [*ctx*, *ace*, *zot* and *tcp*] that is related to intestinal infections are common only with clinical *V*. *mimicus* isolates. This explains in part our result on the prevalence of the virulence genes targeted in the *V*. *mimicus* isolates. Furthermore, the none detection of the *ToxR* gene even in the *OmpU*^+^ *V*. *mimicus* could be explained as earlier discussed for the *V*. *cholerae*. Nevertheless, none of the *V*. *mimicus* could be called avirulent because they all harboured at list one of the virulence determinant targeted in this study. Even if none of the targeted virulent-associated genes is detected in *V*. *mimicus* isolates, the targeted *vmh* gene for the PCR confirmation of the *V*. *mimicus* isolates is a virulence determinant [91]. The gene encodes a heat-labile hemolysin, designated *V*. *mimcus* hemolysin [5, 92] based on their findings suggested the gene as the most important virulence factor of the pathogen. The *toxR*, *zot*, *ctx*, VPI, *ompU*, *rtx* and *hylA* genes have been reported in both clinical and environmental *V*. *mimicus* isolates [4, 65, 93] however, [94] believed that the *tcpA*^*+*^ *V*. *mimicus* strain they reported and which we also isolated in the present study is rare. Our literature search confirmed that the strain is scarce nevertheless, it has been reported by [65] before the report of [94]. The analysis of the result of the virulence determinants in this study and our literature search showed that the peculiarity of virulence determinants among vibrio genus members is gradually diminishing. This supports the position of earlier reports and our study that members of the vibrio genus are evolving in terms of virulence. This evolution might lead to the emergence of pathogenic *Vibrio* spp. that are more virulent than those earlier known.

Our findings in terms of the complex mix of virulence determinants found in non-O1 and non-O139 *V*. *cholerae* strains and recent studies on virulence determinants of non-O1 and non-O139 strains suggested that *V*. *cholerae* is undergoing an evolution that might lead to the emergence of highly virulent strains. Atypical El Tor strains that share characteristics of prototype classical, the El Tor biotypes that is not known to exist before 2002 and strains that cause more severe cholera outbreak has been reported [95–102]. Also, the emergence of SA-NAG *V*. *cholerae* strain that shares genomic [e.g. presence of rtxC, El Tor and the Classical hemolysin gene] and phenotypic attributes of O139 and O1 serogroups have been reported [28]. The detection of the repeat toxin [rtxC] gene which is one of the genomic characteristics that confirms *V*. *cholerae* isolates as of the El Tor biotypes in non-O1 and non-O139 in this study is another pointer to the possible evolution of new *V*. *cholerae* [98, 103–105]. Summarily, the PCR detection of El Tor hemolysin gene in 74%, TcpA ^*El Tor*^ in 11% and rtxC genes in 71% of the non-O1/non-O139 isolates analyzed in this study supports the possibility of the emergence of a typical pathogenic SA-NAG *V*. *cholera* strains that could be more virulence than the known pathogenic strains. This affirms the need for surveillance for non-O1/non-O139 in clinical and non-clinical environments. The need for a surveillance programme for all *Vibrio* spp. in the clinical and environmental settings has been emphasized by a study carried out in Germany [106]. The call for this kind of program is due to the non-specific spread of virulence determinants among the members of the genus as well as the ongoing global warming challenge which is predicted to cause an increase in the abundance of *Vibrio spp*. and their infections in the clinical and non-clinical environment.

### 4.3 Virulence determinants detected in non-O1 and non-O139 *V*. *cholerae* and *V*. *mimicus* isolates and their role in pathogenicity

Eight of the eleven virulence genes targeted in this study were detected and of the eight detected, *toxR*, *rtx* and *hlyA ELTor* virulence genes predominate as earlier reported [65]. The *rtxA* is the most detected of the two *rtx* genes. Similar to earlier studies, some of the isolates in this study possessed both *rtxA* and *rtxC* genes, while neither of the two genes was detected in a few of the isolates [107, 108].

#### 4.3.1 *Vibrio cholerae* non-O1 and non-O139

The predominance of the three genes [*toxR*, *rtx* and hlyAET] in the non-O1 and non-O139 *V*. *cholera* and *V*. *mimicus* speaks to the public health significance of the current study. It has been reported that ctxA− tcpA− zot− stn/sto− but HlyAET+ Non-O1 and non-O139 *V*. *cholerae* can cause diarrhoea and septicemia in humans, and intestinal and extra-intestinal pathophysiological conditions in the animal model [63, 109].

The detection of *rtxA* and *rtxC* in the current study is at per with some earlier studies [28, 65, 108, 110] although a higher prevalence of *rtxA* and *rtxC* is recorded in the current study. The actin cross-linking repeats in the toxin gene cluster [*rtx*] lead to actin depolymerization when cross-linked with Hep-2 and this causes villi effacement, hemorrhagic colitis, and bloody diarrhoea as earlier reported [103, 111]. This suggests the need to screen for the SA-NAG *Vibrio cholerae* in the diarrhoea cases when common etiological agents [e.g. *Escherichia coli* and *Vibrio cholera* O1 and O139 serogroups] are not isolated/detected. It is also needful to screen for this non-O1/non-O139 serogroup when poly-bacterial diarrhoea infection is suspected.

ToxR and HylA^*El Tor*^ are the other two prevalence virulence-associated genes in this study. The prevalence of *toxR* [74.3%] and *hylA* [74.3%] in our study are similar to that of earlier studies [63, 108] from clinical and environmental samples. These two genes are part of the five [*hlyA*, *hlyU*, *hlx*, *toxR* and *attRS1*] virulence-associated genes which were found in the *Vibrio cholerae* non-O1/non-O139 strains that cause cholera epidemics in Calcutta, India. Surprisingly, all the strains isolated from the epidemics lacked the core virulence determinants [*ctx* elements and *tcp* genes] of the toxigenic *V*. *cholerae* O1 and O139 [66]. The regulatory protein ToxR controls the expression of the virulence determinants in *V*. *cholerae* O1, O139 [66] while the product of *hylA* causes haemolysis of erythrocytes [112]. It has been posited that *Vibrio cholerae* non-O1/non-O139 could acquire *tcpA* and *ctxA* which could favour the emergence of novel toxigenic strains under the biological and physicochemical selective pressures of the aquatic environment [63]. The detection of *tcpA* 4/35 [11.4%] and *ace* 1/35 [2.9%] in the current study supports the position of Ottaviani et al. [2018].

The Outer Membrane Protein U [OmpU] gene is another virulence determinant detected in the non-O1/non-O139 strains in this study. This gene is an adherence factor that has been reported in *V*. *cholerae*, *V*. *alginolyticus* and *V*. *mimicus* and it is one of the genes that are responsible for pathogens ability to colonize the intestine [88, 113–115]. The *OmpU* and *OmpT* genes are called pore-forming proteins of the outer membrane and they are regulated by *ToxR* gene [88].

### 4.3 Antibiotics susceptibility patterns of *V*. *cholera and V*. *mimicus* isolates from market and surface water samples

#### 4.3.1 *Vibrio cholerae*

The antibiotics susceptibility pattern observed in this study varies across *Vibrio* spp., sample types, source of isolates and sampling sites. The *V*. *cholerae* isolates were either susceptible or exhibited intermediate susceptibility to amikacin, gentamycin, ofloxacin and cefuroxime. Gentamycin and amikacin interfere with protein synthesis, ofloxacin affect DNA synthesis while cefuroxime interferes with cell wall synthesis. Earlier studies have shown that *V*. *cholerae* non-O1/non-O139 are generally susceptible to the four antibiotics [43, 61, 63, 108, 116–120]. To interpret other susceptibility/resistance patterns for the remaining panel of antibiotics, resistance to an antibiotic was referred to as low to medium when the population that exhibited resistance against it is < 50% [121] and high when the population is > = 50% [122]. Going by this interpretation criteria, the *V*. *cholerae* exhibited high resistance to polymyxin, amoxicillin/clavulanate and ampicillin that target the cell wall synthesis but low/medium resistance to the other eleven panels of antibiotics. The eleven antibiotics that *V*. *cholerae* exhibited low to medium resistance against can be categorized into three. Less than 10% of the *V*. *choloerae* isolates exhibited resistance against the first category which is made up of imipenem, meropenem and cefuroxime; while 10–20% exhibited resistance against the second group which is made up of kanamycin, azithromycin, cefotaxime, chloramphenicol and ciprofloxacin. A greater than 20% but less than 35% of the *V*. *cholerae* isolates exhibited resistance against the third group [nalidixic, nitrofurantoin, cotrimoxazole and trimethoprim]. The high resistance exhibited against polymyxin B and ampicillin has been reported and this seems to be common among environmental isolates of *V*. *cholerae* non-O1/non-O139 strains [108, 123] and O139 toxigenic and non-toxigenic serogroup [124]. The high resistance to polymyxin B and the high prevalence of *hylAElTor* virulence determinant confirms that most of our *V*. *cholerae* isolates belong to El Tor biotype while others might have lost the El Tor marker [polymyxin B resistance] or belong to classical biotype. Polymyxin B resistance is one of the markers for detecting the El Tor biotypes however, it has been reported that the EL Tor biotype can lose this marker [125]. The El Tor strains compare to the classical strains are more adapted and resilient to the environment, cause a higher infection-to-case ratio and have more asymptomatic carriers [95, 126]. Some studies have reported low resistance of the SA-NAG *V*. *cholerae* against amoxicillin/clavulanate [117, 127, 128] however, other studies like ours have shown that different *V*. *cholerae* serotypes can exhibit high resistance against amoxicillin/clavulanate [129, 130].

The low/medium resistance against eleven antibiotics [meropenem, imipenem, cefotaxime, kanamycin, azithromycin, chloramphenicol, ciprofloxacin, nalidixic acid, nitrofurantoin, trimethoprim and cotrimoxazole] by the O1/non-O139 strains recorded in this study is similar to most of the earlier reports [54, 108, 110, 117, 118, 120, 127, 128, 131, 132] although with some differences. A low/medium resistance observed against nalidixic acid by [120, 132], azithromycin by [131], cefotaxime by [132], imipenem by [108, 132], cotrimoxazole by [108, 118, 120, 131, 132], chloramphenicol by [116, 120, 132], ciprofloxacin by [116, 120, 132] and kanamycin by [116, 120] was as observed in this study. However, in some other reports, no *V*. *cholerae* non-O1/non-O139 isolate exhibited resistance against nalidixic acid [110, 117, 128], meropenem [108, 118], cefotaxime [108, 117, 118], imipenem [116–118], nitrofurantoin [108], cotrimoxazole [110, 117], chloramphenicol [108, 110, 117, 118, 131], ciprofloxacin [108, 110, 117, 118], kanamycin [110] and trimethoprim [128]. The percentage of *V*. *cholerae* non-O1/non-O139 strains that exhibited resistance/intermediate status against each of the antibiotics employed for this study was generally high compares to that reported in all the literature consulted.

The antibiotics commonly recommended for the management of vibrio infections fall into four classes of antibiotics [tetracyclines, fluoroquinolones, macrolides, and trimethoprim/sulfamethoxazole] [133–135]. Of the four recommended, three were used in this study and the percentage of *V*. *cholerae* non-O1/non-O139 strains that exhibited resistance against members of the three classes was mostly more than what has been reported in similar studies.

Although the *V*. *cholerae* population in this study exhibited low resistance against kanamycin, meropenem, imipenem, ofloxacin and ciprofloxacin, a high percentage of them exhibited intermediate susceptibility to five antibiotics. This is worrisome because intermediate susceptibility for an antibiotic means a higher dose than recommended is needed for the antibiotic to be effective [136, 137]. The concern stems from the fact that these antibiotics are categorized as critically important antimicrobials for human medicine [138]. The aforementioned suggests that *V*. *cholerae* isolates in this study have the potential to cause cholera-like and vibriosis that might be difficult to manage with commonly recommended and relatively inexpensive antibiotics at the usual dosage. Unfortunately, using higher doses and expensive antibiotics raises resistance, side-effects and economic concerns [139–141].

In light of the aforementioned and the low/medium resistance exhibited by the *V*. *cholerae* population against 80% of the concerned antibiotics, we opined that most of the *V*. *cholerae* isolates in this study have the potential to cause cholera-like and vibriosis that might be difficult to manage with commonly recommended and relatively inexpensive antibiotics.

#### 4.3.2 *Vibrio mimicus*

The phenotypic resistance profile of *V*. *mimicus* population was similar to that of *V*. *cholerae* except for view differences. The differences include: 1. No *V*. *mimicsu* isolate exhibited phenotypic resistance against imipenem, chloramphenicol and ciprofloxacin which *V*. *cholerae* population exhibited low/medium phenotypic resistance against, 2. the *V*. *mimicus* population exhibited low/medium resistance against cefuroxime which none of the *V*. *cholerae* exhibited resistance against, 3. *V*. *mimicus* population exhibited high resistance against nitrofurantoin to which *V*. *cholerae* exhibited low/medium resistance and, 4. *V*. *mimicus* isolates exhibited low/medium resistance against polymyxin B, amoxicillin/clavulanate and ampicillin while the *V*. *cholerae* isolates exhibited high resistance against the three antibiotics. Reports on the antibiotics resistance profile of *V*. *mimicus* isolates are limited in the literature however, few studies we come across during our literature search reported resistance patterns of the organism against all members of the panels of antibiotics used in this study except amikacin [20, 55, 93, 142–144]. Interestingly, all *V*. *mimicus* isolates in this study were susceptible to amikacin. The level of resistance observed against each member of the panel of antibiotics in this study was similar in some instances and differs in others to the earlier reports. High resistance against kanamycin and cotrimoxazole was reported by [142] but no resistance to cotrimoxazole and very low resistance to kanamycin was reported three years later [144]. The current study reports low resistance against the two antibiotics while Gxalo et al., [2021] reported high resistance against kanamycin like Chowdhury et al., [1986]. Also, Nilavan et al., [2021] reported 100% susceptibility, Abdalla et al., [2022] reported low/medium resistance while Raissy et al., [2012] reported high resistance of the environmental *V*. *mimicus* isolates to cotrimoxazole. The medium resistance [44%] against ampicillin reported in the current study was the same as what Chowdhury et al., [1986] reported but differs from the very high resistance [>80%] reported by Abdalla et al., [2022]; Gxalo et al., [2021]; Nilavan et al., [2021]; Raissy et al., [2012] and Yamanaka and Aziz, [1989]. This same scenario goes for other antibiotics used against *V*. *mimicus* in this study and it suggests that local antibiogram result should inform the choice of antibiotics for the management of *V*. *mimicus* infections. In the light of this and considering the intermediate susceptibility result, amikacin, gentamycin and ciprofloxacin are the three antibiotics that can be considered good choice for the management of *V*. *mimicus* infections in our study area although this is subject to other factors such as cost and recommendations of the regulatory bodies such as World Health Organization, Center for Disease Control and Prevention and local regulatory bodies in-charge of our study area.

### 4.4 Genomic bases for the phenotypic antibiotic resistance observed in *V*. *cholerae* and *V*. *mimicus*

A recent systematic review and meta-analysis [145] pointed out the paucity of information on the presence of resistance genes in *Vibrio* spp. The frequency of detection of the twenty-two antibiotics resistance determinants focused on in this study varies for *V*. *cholerae* and *V*. *mimicus*. Forty-three per cent of the *V*. *cholerae* isolates and twenty-two per cent of the *V*. *mimicus* isolates were positive for at least one of the virulence determinants targeted in this study. The *sxt* element which is a conjugative-transposon-like mobile gene element that encodes multiple antibiotic resistance [146] was not detected in this study and this is in line with an earlier report [123]. Phenotypic-genotypic/genotypic-phenotypic resistance discrepancy reported in earlier studies was observed in the current study [147, 148]. Of the six resistance genes [*cmlA*, *cat*, *flor*, *acc*, *ant* and *aphA*] that confer resistance against protein synthesis inhibitors, *cmlA*, *cat*, *flor* that coffer resistance against chloramphenicol [149, 150] were not detected. The *acc* and *ant* that confers resistance against aminoglycosides [amikacin, kanamycin and gentamycin] [151], were detected. However, all isolates that carry the aminoglycosides resistance genes were either phenotypically susceptible or exhibited intermediate susceptibility to the aminoglycosides while one of the isolates surprisingly exhibited resistance against chloramphenicol. The *aphA* that encodes kanamycin resistance [152] was not detected in any of the isolates yet five isolates exhibited phenotypic resistance against kanamycin. Eighty-nine per cent [16/18] of the isolates that exhibited resistance against antifolate drug carries one or both of the *Drf18* and *sul1* gene that encodes antifolate resistance.

The current study supports the few reports that recently detected *mcr-1* gene in *Vibrio* specie [153–155]. One *V*. *cholerae* isolate was positive for *mcr-1* gene which confers resistance to polymyxin E [colistin], polymyxin B and this gene can cause cross-resistance to other antimicrobials [156–159]. This isolate was only positive for one more of the targeted resistance determinants [*gyrB*] but interestingly, it is a multidrug-resistant isolate with MARI of 0.56. This observation could be due to the presence of *mcr-1* gene that causes cross-resistance to other antimicrobial agents in the isolate. Some bacteria which include *V*. *cholerae* are typically resistant to polymyxin [160] however detection of the plasmid-mediated *mcr-1* gene in them raises public health concerns in terms of the spread of colistin resistance [161, 162] because colistin is one of the antibiotics of last resort [157].

The information on the prevalence of β-lactamases in non-O1/non-O139 *Vibrio cholerae* is limited in the literature and we could not find information on the prevalence of β-lactamases in *V*. *mimicus*. Of the eight β-lactamses [*blashv*, *blaoxa*, *blatem*, *blavim*, *blakpc*, *blandm*, *blaimp* and *blaoxa48*] targeted in this study, four [*blashv*, *blaoxa*, *blatem*, *blaoxa48*] were detected and of the four, *blashv* was the most detected while *blaoxa48* was the least detected. All the four detected β-lactamses were found in at least one *V*. *cholerae* while only *blashv* and *blatem* were detected among the *V*. *mimicus* isolates. The *blaoxa*-1 gene was detected in three *V*. *cholerae* isolates and one of the three aslo carries *blaoxa48*. The co-existence of bacteria carrying variants of *blaoxa* genes within the same ecological niche as observed in this study has been reported [163]. We expected the *V*. *cholerae* isolate that was positive for both *blaoxa-48* and *blaoxa-1* resistance genes to exhibit phenotypic resistance against more members of the β-lactam antibiotics used in this study than the two that carry only the *blaoxa-1* because the *blaoxa-1* gene is easily deactivated by penems [164]. Also, we do not expect *blaoxa-*48 positive isolate to exhibit a high level phenotypic resistance against the penems since the *blaoxa-*48 enzyme only hydrolyses penicillins at a high level, broad-spectrum cephalosporins at a moderate level, carbapenems at a low level but not susceptible to β-lactamase inhibitors [165]. However, surprisingly, the two isolates that were negative for the *blaoxa-48 like* but positive for *blaoxa-1* were resistant to all of the β-lactam antibiotics used in this study except imipenem but the *blaoxa-48* and *blaoxa-1* positive isolate exhibited resistance to ampicillin and amoxicillin/clavulanate but susceptible to imipenem, meropenem, cefotaxime and cefuroxime. The *blashv* gene detected in the two *V*. *cholerae* isolates that were positive for only *blaoxa-1* but not detected in the third isolate that was positive for *blaoxa-48* and *blaoxa-1* explains this observation because the association of oxacillnases gene with extended-spectrum β-lactamases [ESBL] genes such as *blashv* often increases the level of resistance to carbapenems [166]. The *blashv* gene is the most detected of all the eight β-lactamases producing genes targeted in this study but isolates harbouring it exhibited varying patterns [six patterns detected as shown in Table 4] of phenotypic resistance to ampicillin, penicillin, amoxicillin/clavulanate, cefuroxime and cefotaxime. Going by the definition of extended β-lactamases, broad spectrum β-lactamases and penicillinases [167–170], we suggest that our isolates carry different variants of *blashv* gene which could belong to any of the 149 fully characterized variants recently reported [168] because we noticed that primer used to target *blashv* is not specific for a particular variant when be BLAST it using National Center for Biotechnology Information PRIMER-BLAST tool. Also, the variants could either belong to the two newly detected variants [*blashv*-230 with accession number NG 148673.1 detected in *E*. *coli* and *blashv*-231 with accession number NG_148674.1 detected in *Klebsiella pneumonia*] which were part of the hits returned by the PRIMER-BLAST. The three isolates with *blatem* gene were resistant to at least one of the penicillin antibiotics used in this study. The *blatem* gene is one of the Class A β-lactamases that exhibit a broad substrate hydrolysis profile against penicillins, cephalosporins, and, for a few of the enzymes, carbapenems [171].

Two phenotypic resistance patterns against β-lactam antibiotics [cefotaxime^R^-cefuroxime^S^ and amoxicillin/clavulanate^R^-ampicillin^S^] observed in this study are strange because we expect a fourth-generation cephalosporin to be more active than third-generation cephalosporin and a penicillin/beta-lactamase inhibitor to be active than penicillin alone. Also, we did not come across these phenotypes in the literature. A research article report and a review [168, 172] have shown that the *blasshv-*mediated phenotypic and genotypic resistance could be so complex to explain at times due to various factors. Summarily the factors as pointed out in literature are: 1. the *balshv* family is highly diverse and complex [a large number of allelic variants including extended-spectrum β-lactamases [ESBL], non-ESBL and several not classified variants exist] in nature, 2. β-lactamase produced in small quantity do not contribute significantly to antibiotic resistance and, 3. overproduction of a β-lactamases could mask the effect of other β-lactamases [168, 173–178]. Furthermore, *Vibrio parahaemolyticus* isolates that are more susceptible to a third-generation cephalosporin [ceftazidime] than fourth-generation cephalosporin [cefepime] have been reported [179]. Also, an unusual case of resistance to amoxicillin/clavulanate but susceptibility to ampicillin in *Enterobacter cloacae* isolate has been reported [180]. However, the mechanism[s] for this rather weird phenotypic resistance pattern remains unexplained in the literature.

The genomic basis for quinolone resistance has been linked to mutation at quinolone resistance-determining regions [QRDRs] of *gyrA*, *gyrB* and *ParC* genes [181]. This region was targeted in this study using primers that are specific for mutation at the QRDRs of the three genes. Unfortunately, we had challenges sequencing the resulting amplicons which is the usual practice. Nevertheless, our result was interpreted using the only four articles found in the National Library of Medicine of the NCIB database that used the same primers we used for their studies [182–185]. Their works showed that mutation at the QRDRs always results in phenotypic resistance to quinolones hence we suspect the occurrence of mutation at the QRDRs of the *gyr* and *par* genes of 88% [15/17] of our isolates that exhibited resistance against the quinolones. Active efflux pump or *qnr* gene that were not targeted in this study could be responsible for the phenotypic resistance against quinolones exhibited by the other 22% [2/17]. Active efflux pump or *qnr* genes have been linked to phenotypic resistance even in the absence of mutation in the *gyr* and *par* genes [185, 186]. It was observed that some isolates harbour resistance genes without corresponding phenotypic resistance. For example, the four *V*. *cholerae* isolates that were *acc* and *ant* positive were susceptible to gentamycin. This kind of phenomenon in which bacterial isolates were susceptible to chloramphenicol despite carrying the chloramphenicol resistance gene has been reported [128].

We noticed a higher rate of phenotypic resistance and detection of resistance genes in isolates from fish samples compared to that from crab, mud prawn and mussel samples. This suggests that *Vibrio* spp. from fish are of more public health significance. We also observed phenotypic resistance in the absence of corresponding resistance genes and the presence of resistance determinants without corresponding phenotypic resistance in isolates. This suggests that our isolates could be engaging other antibiotics resistance mechanisms such as efflux pump [either singly or in synergy with resistance genes] to archive antibiotics resistance. The energy-dependent efflux of multiple antimicrobial agents from bacterial cells of the *Vibrio* spp. is a widely recognized resistance mechanism [187] and some of the specific efflux pumps found in vibrio are EmrD-3, VceB and VcrM Multidrug Efflux Pumps [188].

## 5. Conclusion

The virulence genes, resistance genes and phenotypic resistance heterogeneous profiles observed among our isolates [Table 6] suggest that the three variables [sampling sites, sample types and sources of the samples] got contaminated with *V*. *cholerae* and *V*. *mimicus* from different sources. The group of isolates from these variables have different profiles for resistant genes, virulence genes and phenotypic antibiotics resistance. Interestingly, to corroborate the aforementioned, the virulence genes, resistance genes and phenotypic resistance profiles is unique for each of the *Vibrio* spp. isolates as shown in S3 Table. Also, the report on the heterogeneity of the virulence profile of enteropathogenic *E*. *coli* from different sources supports our opinion [189]. Microbial source tracking coupled with genomic relatedness study on the isolates is expected to unravel this observation in our future study.

Considering the virulence status of our isolates, the relatively high percentages of multidrug-resistant phenotypes [MDRP], [44.44% for *V*. *mimicus* and 65.71% for *V*. *cholerae*], multiple antibiotics resistance phenotypes [MARP] [44.44% for *V*. *mimicus* and 88.57% for *V*. *cholerae*], > 0.2 multiple antibiotics indexes [0.22 for *V*. *cholerae* and 0.21 for *V*. *mimicus*], and the detection of resistance determinants in our isolates, we propose that the environmental sources of our isolates pose a public health risk in terms of the spread of antibiotics resistant strains of *V cholerae* non-O1/non-O139 and *V*. *mimicus* which could cause vibriosis or cholera-like infections that may be difficult to manage with commonly recommended antibiotics. Thus, the findings of this study support the need for prospective surveillance for antibiotic resistance among bacterial isolates from the environment for understanding and minimizing the spread of antibiotic resistance. The CDC recommends regular surveillance for antibiotic resistance among bacterial isolates from any environment as this is essential for understanding and minimizing the spread of resistance [190].

## 6. Highlights

1. The study confirms that fish is one of the main reservoirs of *V*. *cholera* and *V*. *mimicus* in the aquatic environment
2. Eastern Cape Province environment harbours multi-drug resistant and virulent non-O1/non-O139 *V*. *cholerae* and *V*. *mimicus* strains.
3. The observed resistance and virulence profile of isolates in this study calls for the monitoring of environmental non-O1/non-O139 *V*. *cholerae* and *V*. *mimicus*
4. The risk of contracting non-O1/non-O139 *V*. *cholerae* and *V*. *mimicus* infections is higher at the fish markets than at the surface water in Eastern Cape Province, South Africa
5. An inverse relationship was observed between antibiotic resistance and virulence as reported for Non-O1, Non-O139 *Vibrio cholerae* Isolates in an earlier work
6. *OmpU*^*+*^ and *ToxR*^*-*^ strains of non-O1/non-O139 *V*. *cholerae* and the prevalence of β-lactamases genes in *V*, *mimicus* are reported for the first time in this study

## Supporting information

S1 Fig Gel pictures sample showing PCR duplex amplification products of the specific regions of *rtx A* and *rtx C* genes Lane 1 = 100bp molecular marker, lane 2 = negative control, lane 3 = positive control and lanes 4–12 = positive isolates. (TIF)

S2 Fig Gel pictures sample showing PCR duplex amplification products of the specific regions of *toxR* and *ompU* genes Lane 1 = 100bp molecular marker, lane 2 = negative control, lanes 4 &3 = ToxR positive isolates, lanes 5&6 = *ToxR* & *ompU* positive isolates. (TIF)

S3 Fig Gel pictures sample showing PCR sigleplex amplification product of the specific region of *vpi* gene Lane 1 = 100bp molecular marker, lane 2 = negative control, lanes 3,11&12 = negative isolates and lanes 4–10 = positive isolates. (TIF)

S4 Fig Gel pictures sample showing PCR sigleplex amplification product of the specific region of *hylA* gene Lane 1 = 100bp molecular marker, lane 2 = negative control, lane 11 = negative isolate and lanes 3–10; 12&13 = positive isolates. (TIF)

S5 Fig A: Gel pictures sample showing PCR singleplex amplification product of the specific region of *tcp* gene Lane 1 = 100bp molecular marker, lane 2 = negative control, lane 3 = isolate that is negative for tcp gene, lane 4–6 = TCP positive isolates B: Gel pictures sample showing PCR singleplex amplification product of the specific region of ace gene Lane 1 = 100bp molecular marker, lane 2 = positive isolate.
(TIF)

S6 Fig Gel picture showing DNA amplification bands for *BlaSHV*, *BlaOXA* and *BlaTEM* positive DNA templates. Lane 1 = 100bp gene ladder, lanes 6 and 16 = *BlaOXA* positive templates, lanes 16 and 21 = *BlaSHV* positive and *BlaTEM* positive respectively, and other lanes = DNA templates that are not positive for any *BlaSHV*, *BlaOXA* and *BlaTEM* resistance determinants.
(TIF)

S7 Fig Gel pictures showing DNA amplification bands for *sul1*, *ant*, *acc*, *gyrB*, and *ParC* positive DNA templates. A: Lane 1 = 100bp gene ladder, lane = sul 1 positive isolate; B: Lane 1 = 100bp gene ladder, lane 2 = ant positive isolate, lane 3 = acc positive isolate; C: Lane 1 = 100bp gene ladder, lane 3: *parC* positive isolate, lanes 4&5 = *gyrB* positive isolates; every other lane is for templates of isolates that are not positive for any of *sul1*, *ant*, *acc*, *gyrB*, and *ParC* genes.
(TIF)

S8 Fig Gel picture showing DNA amplification bands for *Blaoxa48*, *gyrA*, *Drf18* and *mcr-1* positive DNA templates. A: Lane 1 = 100bp gene ladder, lanes 4&5 = gyrA positive isolates; lane 6 = mcr-1 positive isolate; B: Lane 1 = 100bp gene ladder, lanes 2&4 = dfr18 positive isolates, lane 6 = *BlaOXA48* positive isolate.
(TIF)

S1 Table List of primers for the confirmation *V*. *cholerae* serotype and detection of virulence determinants. Key: AMS = amplicon size, Ref = references.
(DOCX)

S2 Table Primer list for the detection of antibiotics resistance determinants [PCR conditions are as reported in the references of the primers]. Key: AMS = amplicon size, Ref = references.
(DOCX)

S3 Table Distribution of resistance genes, phenotypic resistance and virulence genes combinations among *V*. *cholerae* [n = 34] and *V*. *mimicus* [n = 10] population. Key: Vc = *Vibrio cholera*, Vm = *Vibrio mimicus*, Black color = Vc only, Blue colour = Vm Only, Red colour = both.
(DOCX)

S4 Table Dynamics of resistance for sampling sites, sample types, sources of samples and class of sample types using MARI, MARP AND MDRP as indices. Key: n = number of isolates, MARI = multiple antibiotics resistance index, MARP = multiple-antibiotics resistance phenotype, MDRP-Multiple-drug resistance phenotype, Brac-water = brackish water, Crustan = Crustaceans.
(DOCX)
