# Supplementary figures and images for: Antibiotic resistance and virulence genes profiling of Vibrio cholerae and Vibrio mimicus isolates from some seafood collected at the aquatic environment and wet markets in Eastern Cape Province, South Africa
Source: PLoS One. 2023 Aug 24;18(8):e0290356. doi: 10.1371/journal.pone.0290356 (PMC10449182; doi:10.1371/journal.pone.0290356)

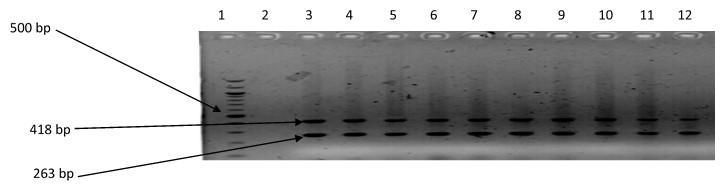

Supplement: S1 Fig — (TIF) [file pone.0290356.s001.tif]

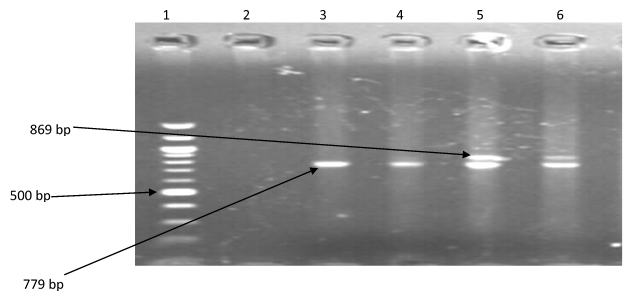

Supplement: S2 Fig — (TIF) [file pone.0290356.s002.tif]

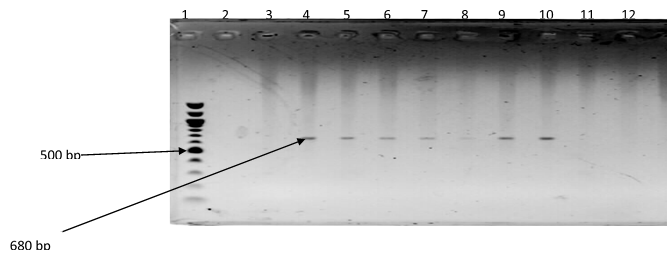

Supplement: S3 Fig — (TIF) [file pone.0290356.s003.tif]

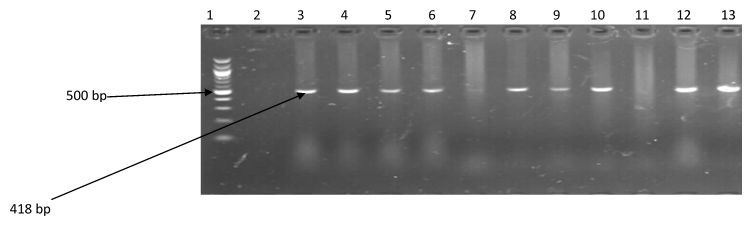

Supplement: S4 Fig — (TIF) [file pone.0290356.s004.tif]

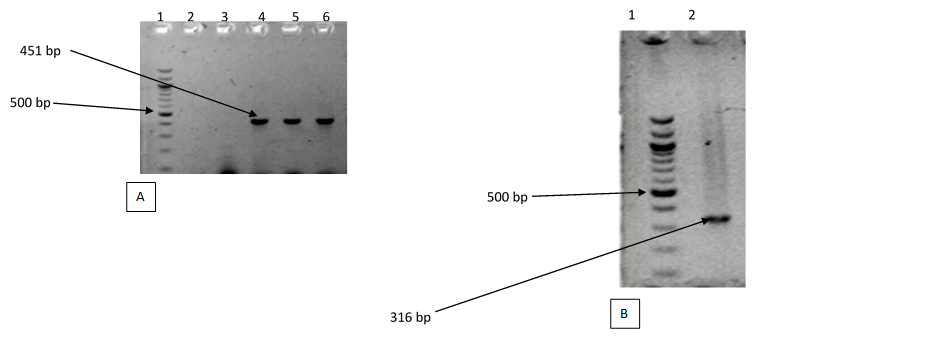

Supplement: S5 Fig — A: Gel pictures sample showing PCR singleplex amplification product of the specific region of tcp gene Lane 1 = 100bp molecular marker, lane 2 = negative control, lane 3 = isolate that is negative for tcp gene, lane 4–6 = TCP positive isolates B: Gel pictures sample showing PCR singleplex amplification product of the specific region of ace gene Lane 1 = 100bp molecular marker, lane 2 = positive isolate. (TIF) [file pone.0290356.s005.tif]

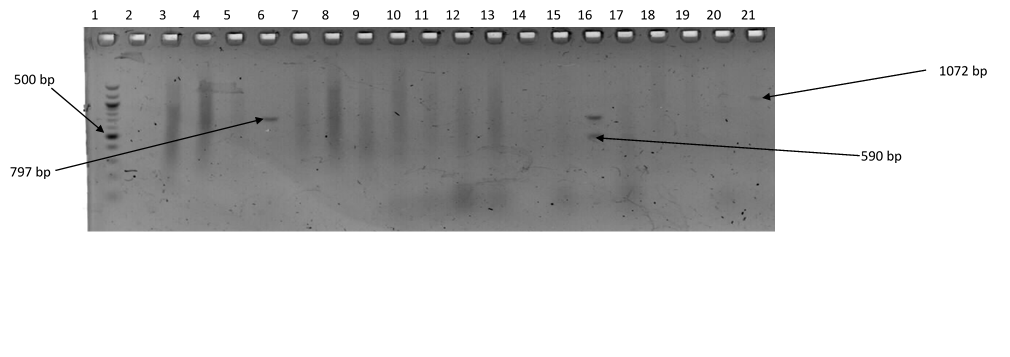

Supplement: S6 Fig — Lane 1 = 100bp gene ladder, lanes 6 and 16 = BlaOXA positive templates, lanes 16 and 21 = BlaSHV positive and BlaTEM positive respectively, and other lanes = DNA templates that are not positive for any BlaSHV, BlaOXA and BlaTEM resistance determinants. (TIF) [file pone.0290356.s006.tif]

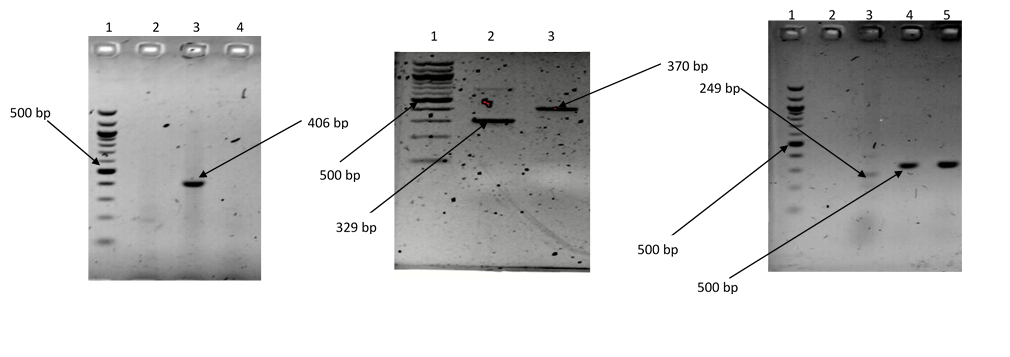

Supplement: S7 Fig — A: Lane 1 = 100bp gene ladder, lane = sul 1 positive isolate; B: Lane 1 = 100bp gene ladder, lane 2 = ant positive isolate, lane 3 = acc positive isolate; C: Lane 1 = 100bp gene ladder, lane 3: parC positive isolate, lanes 4&5 = gyrB positive isolates; every other lane is for templates of isolates that are not positive for any of sul1, ant, acc, gyrB, and ParC genes. (TIF) [file pone.0290356.s007.tif]

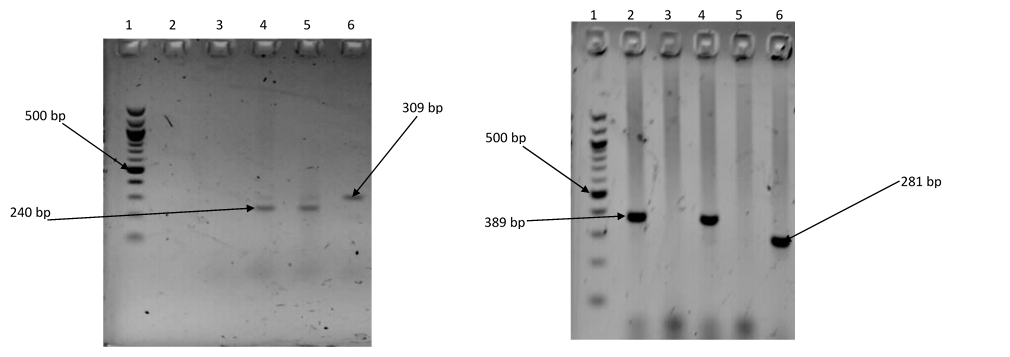

Supplement: S8 Fig — A: Lane 1 = 100bp gene ladder, lanes 4&5 = gyrA positive isolates; lane 6 = mcr-1 positive isolate; B: Lane 1 = 100bp gene ladder, lanes 2&4 = dfr18 positive isolates, lane 6 = BlaOXA48 positive isolate. (TIF) [file pone.0290356.s008.tif]
