# Supplementary material for: Antibiotic resistance and virulence genes profiling of Vibrio cholerae and Vibrio mimicus isolates from some seafood collected at the aquatic environment and wet markets in Eastern Cape Province, South Africa
Source: PLoS One. 2023 Aug 24;18(8):e0290356. doi: 10.1371/journal.pone.0290356 (PMC10449182; doi:10.1371/journal.pone.0290356)
Supplement: S1 Table — Key: AMS = amplicon size, Ref = references. (DOCX) [file pone.0290356.s009.docx]

Table 1: List of primers for the confirmation *V. cholerae* serotype and detection of virulence determinants

| **Confirmation of *V. cholerae* isolates as O1, O139 non-O1/non-O139 serogroup** | | | | | |
| --- | --- | --- | --- | --- | --- |
| **Genes** | **Gene Products** | **Primer's sequence** | **AMS (bp)** | **Tm** (⁰C) | **Ref** |
|  |  |  |  |  |  |
| *O1-rfb* | O1 specific LPS | F:5'-GTTTCACTGAACAGATGGG-3' | 192 | 58.01 | Hoshino et al., 1998 |
|  |  | R:5'-GGTCATCTGTAAGTACAAC-3' |  | 55.85 |  |
| *O139-rfb* | O139 specific LPS | F:5'-AGCCTCTTTATTACGGGTGG-3' | 449 | 60.4 |  |
|  |  | R:5'-GTCAAACCCGATCGTAAAGG-3' |  | 60.4 |  |
| *Vibrio* genus | *Vibrio* spp. F | F:5'-CGG TGA AAT GCG TAG AGA T-3' | 663 | 58.01 |  |
|  | *Vibrio* spp. R | R:5'-TTA CTA GCG ATT CCG AGT TC-3' |  | 58.35 |  |
| **Detection of virulence determinants** | | | | | |
| **Gene** | **Gene Products** | **Primer's sequence** | **AMS (bp)** | **Tm** (⁰C) | **Ref** |
|  |  |  |  |  |  |
| *rtxA* | repeat like toxin subunit A | F:5′-CTG AAT ATG AGT GGG TGA CTT ACG-3′ | 418 | 62.86 | Chow et al 2001 |
|  |  | R:5′GTG TAT TGT TCG ATA TCC GCT ACG-3′ |  | 62.86 |  |
| *rtxC* | repeat like toxin subunit C | F:5′-CGA CGA AGA TCA TTG ACG AC-3′ | 263 | 60.4 |  |
|  |  | R:5′-CAT CGT CGT TAT GTG GTT GC-3′ |  | 60.4 |  |
| *toxR* | transmembrane transcriptional activator | F:5′-CCTTCGATCCCCTAAGCAATAC-3′ | 779 | 69.69 | Rivera 2001 |
|  |  | R:5′-AGGGTTAGCAACGATGCGTAAG-3′ |  | 64.58 |  |
| *ompU* | pore-forming proteins of the outer membrane | F:5′-ACGCTGACGGAATCAACCAAAG-3′ | 869 | 62.67 | Rivera 2001 |
|  |  | R:5′-GCGGAAGTTTGGCTTGAAGTAG-3′ |  | 62.67 |  |
| *ctxA* | cholera toxin subunit A | F:5’-CTC AGA CGG GAT TGT TAG GCA CG-3’ | 301 | 66.28 | Shirai et al 1991 |
|  |  | R:5’-TCT ATC TCT GTA GCC CCT ATT ACG-3’ |  | 62.86 |  |
| *ctxB* | repeat like toxin subunit B | F:5′-GAT ACA CAT AAT AGA ATT AAG GAT G-3′ | 461 | 56.38 | Olsvik1993 |
|  |  | R:5′-GGT TGC TTC TCA TCA TCG AAC CAC-3′ |  | 64.57 |  |
| *vpi* | vibrio pathogenicity iceland | F:5′-GCA ATT TAG GGG CGC GAC GT-3′ | 680 | 64.5 | Sechi et al 2000 |
|  |  | R:5′-CCG CTC TTT CTT GAT CTG GTA G-3′ |  | 62.67 |  |
| *hylA El Tor* | haemolysin | F: 5′-GAGCCGGCATTCATCTGAAT-3′ | 481 | 60.4 | Rivera 2001 |
|  |  | R: 5′-CTCAGCGGGCTAATACGGTTTA-3′ |  | 62.67 |  |
| *tcpA El Tor* | Toxin co-regulated pili | F: 5′-CAC GAT AAG AAA ACC GGT CAA GAG-3′ | 451 | 62.86 | Rivera 2001 |
|  |  | R: 5′-CGA AAG CAC CTT CTT TCA CGT TG-3′ |  | 62.77 |  |
| *Zot* | zonula occludens toxin | F: 5′-TCGCTTAACGATGGCGCGTTTT-3′  R: 5′- AACCCCGTTTCACTTCTACCCA-3′ | 947 | 62.67 | Menezes *et al .,* 2014 |
|  |  |  |  | 62.67 |  |
| *ace* | accessory cholera enterotoxin | F:5′-TAAGGATGTGCTTATGATGGACACCC-3′  R:5′-CGTGATGAATAAAGATACTCATAGG-3′ | 316 | 64.59 | (Singh et al., 2002) |
|  |  |  |  | 59.66 |  |

Key: AMS=amplicon size, Ref=references
