# Supplementary material for: Antibiotic resistance and virulence genes profiling of Vibrio cholerae and Vibrio mimicus isolates from some seafood collected at the aquatic environment and wet markets in Eastern Cape Province, South Africa
Source: PLoS One. 2023 Aug 24;18(8):e0290356. doi: 10.1371/journal.pone.0290356 (PMC10449182; doi:10.1371/journal.pone.0290356)
Supplement: S2 Table — Key: AMS = amplicon size, Ref = references. (DOCX) [file pone.0290356.s010.docx]

**S1 Table 2: Primer list for the detection of antibiotics resistance determinants (PCR conditions is as reported in the references of the primers)**

| S/N | Resistance Gene Prime Codes | Primer sequences | Expected amplicon sizes (bp) | Tm (⁰C) | Primer references |
| --- | --- | --- | --- | --- | --- |
| **1** | **Drf 18 F** | **TGGGTAAGACACTCGTCATGGG** | 389 | 61.73 | (Mohapatra et al., 2008) |
|  | **Drf 18 R** | **ACTGCCGTTTTCGATAATGTGG** |  | 59.58 |  |
| **2** | **acc F** | **TGAAACGCTGACGGAGCCT** | 370 | 61.87 | (Iwanaga et al., 2004) |
|  | **acc R** | **GTCGAACAGGTAGCACTGAG** |  | 58.02 |  |
| **3** | **blaSHV F** | **TTATCTCCCTGTTAGCCACC** | 797 | 56.31 | (Arlet et al., 1997) |
|  | **blaSHV R** | **GATTTGCTGATTTCGCTCGG** |  | 57.9 |  |
| **4** | **blaOXA f** | **ACCAGATTCAACTTTCAA** | 590 | 48.64 | (Wu et al., 2013) |
|  | **blaOXA R** | **TCTTGGCTTTTATGCTTG** |  | 50.46 |  |
| **5** | **blaTEM F** | **ATAAAATTCTTGAAGAC** | 1072 | 40.4 | (Al Dawodeyah et al., 2018) |
|  | **blaTEM R** | **TTACCAATGCTTAATCA** |  | 44.49 |  |
| **6** | **aphA F** | **GGGACCACCTATGATGTGGAACG** | 595 | 62.86 | (Gibreel et al., 2004) |
|  | **aphA R** | **CAGGCTTGATCCCCAGTAAGTC** |  | 60.42 |  |
| **7** | **Sul 1 F** | **TGAGATCAGACGTATTGCGC** | 406 | 58.16 | (Oliveira et al., 2023) |
|  | **Sul 1 R** | **TTGAAGGTTCGACAGCACGT** |  | 60.18 |  |
| **8** | **Sul 2 F** | **GCGCTCAAGGCAGATGGCATT** | 285 | 64.23 | (Oliveira et al., 2023) |
|  | **Sul 2 R** | **GCGTTTGATACCGGCACCCGT** |  | 65.85 |  |
| **9** | **CmlA F** | **TACTCGGATCCATGCTGGCC** | 578 | 61.76 | (Ravi et al., 2022) |
|  | **CmlA R** | **TCCTCGAAGAGCGCCATTGG** |  | 62.58 |  |
| **10** | **CAT 1F** | **CGCCTGATGAATGCTCATCCG** | 457 | 61.79 | (Aarestrup et al., 2003) |
|  | **CAT 2R** | **CCTGCCACTCATCGCAGTAC** |  | 60.81 |  |
| **11** | **SXTF** | **TCGGGTATCGCCCAAGGGCA** | 946 | 65.97 | (Xiao et al., 2022) |
|  | **SXTR** | **GCGAAGATCATGCATAGACC** |  | 56.47 |  |
| **12** | **FLOR F** | **GTGATTTTTGGTCCGCTCTC** | 739 | 57.1 | (Marin et al., 2013) |
|  | **FLOR R** | **TCGGTAGGATGAAGGTGAGG** |  | 58.22 |  |
| **13** | **gyrA_vF** | **AATGTGCTGGGCAACGACTGG** | 240 | 63.78 | (Marin et al., 2013) |
|  | **gyrA_vR** | **GTGCGCGATTTTCGACATACG** |  | 60.91 |  |
| **14** | **gyrB_vF** | **GGAAATGACTCGCCGTAAAGG** | 310 | 59.34 | (Marin et al., 2013) |
|  | **gyrB_vR** | **GTTGTGATAACGCAGTTTATCTGGG** |  | 60.45 |  |
| **15** | **ParC_vF** | **GTCTGAGTTGGGTCTCTCGGC** | 249 | 62.42 | (Marin et al., 2013) |
|  | **ParC_vR** | **AGAATCTCGGCAAACTTTGACAG** |  | 59.5 |  |
| **16** | **mcr-1 F** | **CGGTCAGTCCGTTTGTTC** | 309 | 56.08 | (Ilbeigi et al., 2021) |
|  | **mcr-1 R** | **CTTGGTCGGTCTGTAGGG** |  | 56.35 |  |
| **17** | **BLAvim F** | GATGGTGTTTGGTCGCATA | 390 | 56.61 | (Shirani et al., 2016) |
|  | **BLAvim R** | CGAATGCGCAGCACCAG |  | 59.54 |  |
| **18** | **BLAkpc F** | CATTCAAGGGCTTTCTTGCTGC | 538 | 60.93 | (Mushi et al., 2014) |
|  | **BLAkpc R** | ACGACGGCATAGTCATTTGC |  | 58.99 |  |
| **19** | **BLAndm F** | GGTTTGGCGATCTGGTTTTC | 621 | 57.65 | (Mushi et al., 2014) |
|  | **BLAndm R** | CGGAATGGCTCATCACGATC |  | 58.86 |  |
| **20** | **BLAimp F** | TTGACACTCCATTTACAG | 139 | 48.98 | (Mushi et al., 2014) |
|  | **BLAimp R** | GATTGAGAATTAAGCCACTCT |  | 53.32 |  |
| **21** | **BLAoxa48 F** | GCTTGATCGCCCTCGATT | 281 | 57.55 | (Mushi et al., 2014) |
|  | **BLAoxa48 R** | GATTTGCTCCGTGGCCGAAA |  | 61.58 |  |
| **22** | **ant F** | GGG CGC GTC ATG GAG GAG TT | 329 | 64.98 | (Cameron et al., 1986) |
|  | **ant R** | TAT CGC GAC CTG AAA GCG GC |  | 63.22 |  |

Key: AMS=amplicon size, Ref=references
