# Supplementary material for: Antibiotic resistance and virulence genes profiling of Vibrio cholerae and Vibrio mimicus isolates from some seafood collected at the aquatic environment and wet markets in Eastern Cape Province, South Africa
Source: PLoS One. 2023 Aug 24;18(8):e0290356. doi: 10.1371/journal.pone.0290356 (PMC10449182; doi:10.1371/journal.pone.0290356)
Supplement: S3 Table — Key: Vc = Vibrio cholera, Vm = Vibrio mimicus, Black color = Vc only, Blue colour = Vm Only, Red colour = both. (DOCX) [file pone.0290356.s011.docx]

S1 Table 3: Distribution of resistance genes, phenotypic resistance and virulence genes combinations among *V. cholerae* (n = 34) and *V. mimicus* (n = 10) population.

| **Resistance Genes profiles** | **Vc (n)** | **Vm (n)** | **Phenotypic resistance profile** | **Vc(n)** | **Vm (n)** | **Viurlence genes profiles** | **Vc(n)** | **Vm (n)** |
| --- | --- | --- | --- | --- | --- | --- | --- | --- |
| Drf 18, gyrA | 2 |  | AK,A,C,AP,TM | 1 |  | hyla | 2 | 3 |
| Drf 18, gyrA, gyrB | 1 |  | AK,TS,C,AP,TM | 1 |  | hyla, rtxA, rtxB |  | 3 |
| Drf 18, gyrA, ParC | 1 |  | AP | 1 | 2 | OmpU, hyla, rtxA, rtxB |  | 1 |
| Gent ant | 3 |  | ATH,PB,NI |  | 2 | OmpU, hyla,rtxA, rtxC | 5 |  |
| Gent ant, Drf18, blasSHV, gyrA, gyrB | 1 |  | ATH,PB,NI,TS,C,AUG,AP,TM | 2 |  | tcp, hyla, rtxA, rtxB |  | 1 |
| gyrA | 1 | 1 | AUG,AP,TM | 2 |  | tcp, rtxA | 1 |  |
| gyrA, gyrB |  | 1 | CTX,AP,TM | 1 |  | tcp, rtxA, rtxC | 1 |  |
| gyrA, ParC | 3 |  | IMI,PB,AP | 1 |  | toxR, hyla, rtxA, rtxC | 3 |  |
| gyrB, CLR | 1 |  | IMI,PB,AUG | 1 |  | toxR, OmpU, hyla,rtxA, rtxC | 7 |  |
| ParC | 2 |  | K,CTX,PB,TS | 3 |  | toxR, tcp,ace,hyla,rtxA, rtxC | 2 |  |
| sul1, gyrA, ParC | 1 |  | K,NA,MEM,ATH,CTX,PB,NI,TS,CXM,AUG,AP,TM | | 2 | vpi, OmpU, hyla, rtxA |  | 1 |
|  |  |  | NA |  | 1 | vpi, toxR, hyla, rtxA, rtxC | 5 |  |
| Vc= *V. cholerae* |  |  | NA,ATH,CTX,PB,NI,TS,AUG,AP,TM | 1 |  | vpi, toxR, OmpU, hyla,rtxA, rtxC | 1 |  |
| *Vm= V. mimicus* |  |  | NA,MEM,ATH,IMI,PB,NI,TS,AUG,AP,TM | 2 |  | vpi, toxR, tcp, hyla,rtxA, rtxC | 1 |  |
|  |  |  | NA,PB,AUG | 2 |  |  |  |  |
|  |  |  | NA,TS,C,AP,TM | 2 |  |  |  |  |
|  |  |  | NI |  | 1 |  |  |  |
|  |  |  | PB,AP | 2 |  |  |  |  |
|  |  |  | PB,AUG | 3 |  |  |  |  |
|  |  |  | PB,CIP,AUG,AP | 5 |  |  |  |  |
|  |  |  | PB,NI,TS,AUG | 2 |  |  |  |  |

Key: Vc = *Vibrio cholera*, Vm = *Vibrio mimicus*, **Black color =Vc only, Blue colour = Vm Only, Red colour = both**
