# Supplementary material for: Antibiotic resistance and virulence genes profiling of Vibrio cholerae and Vibrio mimicus isolates from some seafood collected at the aquatic environment and wet markets in Eastern Cape Province, South Africa
Source: PLoS One. 2023 Aug 24;18(8):e0290356. doi: 10.1371/journal.pone.0290356 (PMC10449182; doi:10.1371/journal.pone.0290356)
Supplement: S4 Table — Key: n = number of isolates, MARI = multiple antibiotics resistance index, MARP = multiple-antibiotics resistance phenotype, MDRP-Multiple-drug resistance phenotype, Brac-water = brackish water, Crustan = Crustaceans. (DOCX) [file pone.0290356.s012.docx]

S1 Table 4: Dynamics of resistance for sampling sites, sample types, sources of samples and class of sample types using MARI, MARP AND MDRP as indices

| ***V. cholerae*** | | | | | | | | | | | | | | | | | | | | | | | | | | | | | | | |  |  |  |
| --- | --- | --- | --- | --- | --- | --- | --- | --- | --- | --- | --- | --- | --- | --- | --- | --- | --- | --- | --- | --- | --- | --- | --- | --- | --- | --- | --- | --- | --- | --- | --- | --- | --- | --- |
| **Sampling Sites** | | | | | | | **Sample types** | | | | | | | **Sources of samples** | | | | | | | **Class of sample types** | | | | | | | | | | |  |  |  |
| **Site (n)** | **MARI** | | **MARP (%)** | | **MDRP (%)** | | **ST**  **(n)** | **MARI** | | **MARP (%)** | | **MDRP (%)** | | **Sources**  **(n)** | **MARI** | | **MARP (%)** | | **MDRP**  **(%)** | | | **Class (n)** | | **MARI** | | **MARP (%)** | | | **MDRP**  **(%)** | | | |  |  |
| ALD2 (2) | | 0.50 | | 100 | | 100 | CR (9) | | 0.36 | | 89 | | 33 | Brac-water (11) | | 0.32 | | 100 | | 36 | | | Fish (22) | | 0.22 | | 86 | | | 86 | | | | |
| EL6 (5) | | 0.38 | | 100 | | 100 | FL (8) | | 0.13 | | 75 | | 75 | Freshwater (6) | | 0.13 | | 83 | | 83 | | | Crustan (12) | | 0.15 | | 92 | | | 42 | | | | |
| PA6 (5) | | 0.16 | | 80 | | 60 | FN (8) | | 0.22 | | 88 | | 88 | Market  (18) | | 0.19 | | 83 | | 83 | | | Mollusc (1) | | 0.11 | | 100 | | | 0 | | | | |
| PA7 (16) | | 0.18 | | 81 | | 69 | G (4) | | 0.15 | | 100 | | 100 |  | |  | |  | |  | | |  | |  | |  | | |  | | | | |
| SKR (7) | | 0.16 | | 100 | | 43 | IN (2) | | 0.11 | | 100 | | 100 |  | |  | |  | |  | | |  | |  | |  | | |  | | | | |
|  | |  | |  | |  | MP (3) | | 0.19 | | 100 | | 67 |  | |  | |  | |  | | |  | |  | |  | | |  | | | | |
|  | |  | |  | |  | MU (1) | | 0.11 | | 100 | | 0 |  | |  | |  | |  | | |  | |  | |  | | |  | | | | |
| ***V. mimicus*** | | | | | | | | | | | | | | | | | | | | | | | | | | | | | | | | | |  |
| **Sampling Sites** | | | | | | | **Sample types** | | | | | | | **Sources of samples** | | | | | | | | **Class of sample types** | | | | | | | | | | | |  |
| **Site (n)** | | **MARI** | | **MARP (%)** | | **MDRP (%)** | **ST (n)** | | **MARI** | | **MARP (%)** | | **MDRP (%)** | **Sources (n)** | | **MARI** | | **MARP (%)** | | **MDRP (%)** | | | **Class**  **(n)** | | **MARI** | | | **MARP (%)** | | | **MDRP**  **(%)** | | |  |
| ALD2 (1) | | 0.06 | | 0 | | 0 | CR (2) | | 0.03 | | 0 | | 0 | Brac-water (5) | | 0.07 | | 20 | | 20 | | | Crustan (2) | | 0.03 | | | 0 | | | 0 | | |  |
| EL6 (2) | | 0.67 | | 100 | | 100 | G (1) | | 0.17 | | 100 | | 100 | Freshwater (1) | | 0.06 | | 0 | | 0 | | | Fish  (4) | | 0.39 | | | 75 | | | 75 | | |  |
| PA6 (2) | | 0.03 | | 50 | | 0 | IN (3) | | 0.46 | | 67 | | 67 | Market  (3) | | 0.50 | | 100 | | 100 | | | Mollusc (3) | | 0.09 | | | 33 | | | 33 | | |  |
| PA7 (4) | | 0.11 | | 50 | | 50 | MU (3) | | 0.09 | | 33 | | 33 |  | |  | |  | |  | | |  | |  | | |  | | |  | | |  |

Key: n = number of isolates, MARI = multiple antibiotics resistance index, MARP = multiple-antibiotics resistance phenotype, MDRP-Multiple-drug resistance phenotype, Brac-water = brackish water, Crustan = Crustaceans

**References for supporting document**

Aarestrup, F.M., Lertworapreecha, M., Evans, M.C., Bangtrakulnonth, A., Chalermchaikit, T., Hendriksen, R.S., Wegener, H.C., 2003. Antimicrobial susceptibility and occurrence of resistance genes among Salmonella enterica serovar Weltevreden from different countries. Journal of Antimicrobial Chemotherapy 52, 715–718. https://doi.org/10.1093/jac/dkg426

Al Dawodeyah, H.Y., Obeidat, N., Abu-Qatouseh, L.F., Shehabi, A.A., 2018. Antimicrobial resistance and putative virulence genes of Pseudomonas aeruginosa isolates from patients with respiratory tract infection. Germs 8, 31–40. https://doi.org/10.18683/germs.2018.1130

Arlet, G., Rouveau, M., Philippon, A., 1997. Substitution of alanine for aspartate at position 179 in the SHV-6 extended-spectrum β-lactamase. FEMS Microbiology Letters 152, 163–167. https://doi.org/10.1111/j.1574-6968.1997.tb10423.x

Cameron, F.H., Groot Obbink, D.J., Ackerman, V.P., Hall, R.M., 1986. Nucleotide sequence of the AAD(2’’) aminoglycoside adenylyltransferase determinant aadB. Evolutionary relationship of this region with those surrounding aadA in R538-1 and dhfrII in R388. Nucleic Acids Res 14, 8625–8635. https://doi.org/10.1093/nar/14.21.8625

Gibreel, A., Sköld, O., Taylor, D.E., 2004. Characterization of plasmid-mediated aphA-3 kanamycin resistance in Campylobacter jejuni. Microb Drug Resist 10, 98–105. https://doi.org/10.1089/1076629041310127

Ilbeigi, K., Askari Badouei, M., Vaezi, H., Zaheri, H., Aghasharif, S., Kafshdouzan, K., 2021. Molecular survey of mcr1 and mcr2 plasmid mediated colistin resistance genes in Escherichia coli isolates of animal origin in Iran. BMC Research Notes 14, 107. https://doi.org/10.1186/s13104-021-05519-6

Iwanaga, M., Toma, C., Miyazato, T., Insisiengmay, S., Nakasone, N., Ehara, M., 2004. Antibiotic Resistance Conferred by a Class I Integron and SXT Constin in Vibrio cholerae O1 Strains Isolated in Laos. Antimicrob Agents Chemother 48, 2364–2369. https://doi.org/10.1128/AAC.48.7.2364-2369.2004

Marin, M.A., Thompson, C.C., Freitas, F.S., Fonseca, E.L., Aboderin, A.O., Zailani, S.B., Quartey, N.K.E., Okeke, I.N., Vicente, A.C.P., 2013. Cholera Outbreaks in Nigeria Are Associated with Multidrug Resistant Atypical El Tor and Non-O1/Non-O139 Vibrio cholerae. PLoS Negl Trop Dis 7, e2049. https://doi.org/10.1371/journal.pntd.0002049

Mohapatra, H., Mohapatra, S.S., Mantri, C.K., Colwell, R.R., Singh, D.V., 2008. Vibrio cholerae non-O1, non-O139 strains isolated before 1992 from Varanasi, India are multiple drug resistant, contain intSXT, dfr18 and aadA5 genes. Environmental Microbiology 10, 866–873. https://doi.org/10.1111/j.1462-2920.2007.01502.x

Mushi, M.F., Mshana, S.E., Imirzalioglu, C., Bwanga, F., 2014. Carbapenemase Genes among Multidrug Resistant Gram Negative Clinical Isolates from a Tertiary Hospital in Mwanza, Tanzania. Biomed Res Int 2014, 303104. https://doi.org/10.1155/2014/303104

Oliveira, M., Truchado, P., Cordero-García, R., Gil, M.I., Soler, M.A., Rancaño, A., García, F., Álvarez-Ordóñez, A., Allende, A., 2023. Surveillance on ESBL-Escherichia coli and Indicator ARG in Wastewater and Reclaimed Water of Four Regions of Spain: Impact of Different Disinfection Treatments. Antibiotics 12, 400. https://doi.org/10.3390/antibiotics12020400

Ravi, N.K., Pal, A.K., Soni, R., Tripathi, P., Singhal, A., Jha, P.K., Tripathi, V., 2022. Assessment of antibiotic resistance profile of bacteria isolated from Ghaghara River, India. Water Supply 22. https://doi.org/10.2166/ws.2022.365

Shirani, K., Ataei, B., Roshandel, F., 2016. Antibiotic resistance pattern and evaluation of metallo-beta lactamase genes (VIM and IMP) in Pseudomonas aeruginosa strains producing MBL enzyme, isolated from patients with secondary immunodeficiency. Adv Biomed Res 5, 124. https://doi.org/10.4103/2277-9175.186986

Singh, D. V, Isac, S.R., Colwell, R.R., 2002. Development of a Hexaplex PCR Assay for Rapid Detection of Virulence and Regulatory Genes in Vibrio cholerae and Vibrio mimicus 40, 4321–4324. https://doi.org/10.1128/JCM.40.11.4321

Wu, H., Xia, X., Cui, Y., Hu, Y., Xi, M., Wang, X., Shi, X., Wang, D., Meng, J., Yang, B., 2013. Prevalence of Extended-Spectrum β-Lactamase–Producing Salmonella on Retail Chicken in Six Provinces and Two National Cities in the People’s Republic of China. Journal of Food Protection 76, 2040–2044. https://doi.org/10.4315/0362-028X.JFP-13-224

Xiao, Y., Huang, Z., Yu, K., Wang, M., Gao, H., Bai, X., Jiang, M., Wang, D., 2022. Distribution and Molecular Characteristics of Vibrio Species Isolated from Aquatic Environments in China, 2020. Microorganisms 10, 2007. https://doi.org/10.3390/microorganisms10102007
